# Expansion of Colorectal Cancer Biomarkers Based on Gut Bacteria and Viruses

**DOI:** 10.3390/cancers14194662

**Published:** 2022-09-25

**Authors:** Jia Zhang, Yangting He, Lu Xia, Jing Yi, Zhen Wang, Yingying Zhao, Xuemei Song, Jia Li, Hongli Liu, Xinjun Liang, Shaofa Nie, Li Liu

**Affiliations:** 1 Department of Epidemiology and Biostatistics, Ministry of Education Key Lab of Environment and Health, School of Public Health, Tongji Medical College, Huazhong University of Science and Technology, Wuhan 430030, China; 2 Cancer Center, Union Hospital, Tongji Medical College, Huazhong University of Science and Technology, Wuhan 430023, China; 3 Department of Medical Oncology, Tongji Medical College, Hubei Cancer Hospital, Huazhong University of Science and Technology, Wuhan 430079, China; 4 Colorectal Cancer Clinical Research Center of Hubei Province, Wuhan 430079, China; 5 Colorectal Cancer Clinical Research Center of Wuhan, Wuhan 430079, China

**Keywords:** colorectal cancer, gut microbiome, diagnostic model, bacteria, virus

## Abstract

**Simple Summary:**

The current study identified microbial (including bacterial and viral) diagnostic models that could discriminate colorectal tumor patients from healthy controls, expanding the potential biomarkers for colorectal tumors. A combination of five colorectal cancer-associated gut bacteria was identified in this study for the discrimination of colorectal cancer patients from healthy controls, with verifiable performance in multiple cohorts. The gene pathways regulated by aberrant gut bacteria were also identified, providing possible directions for studying bacterial carcinogenesis mechanisms. Furthermore, this study revealed the potential interactions of gut bacteria with viruses and within bacteria in adenoma-carcinoma sequences, which may extend our understanding of dysbiosis in colorectal carcinogenesis.

**Abstract:**

The alterations in gut bacteria are closely related to colorectal cancer. However, studies on adenoma are still scarce. Besides, the associations of gut viruses with colorectal tumor, and the interactions of bacteria with viruses in colorectal tumors are still under exploration. Therefore, a metagenomic sequencing of stool samples from patients with colorectal adenoma (CRA), colorectal cancer (CRC), and healthy controls was performed to identify changes in gut microbiome in patients with colorectal tumors. Five CRC-enriched bacteria (*Peptostreptococcus stomatis*, *Clostridium symbiosum*, *Hungatella hathewayi*, *Parvimonas micra*, and *Gemella morbillorum*) were identified as a diagnostic model to identify CRC patients, and the efficacy of the diagnostic model was verifiable in 1523 metagenomic samples from ten cohorts of eight different countries. We identified the positive association of *Bacteroides fragilis* with PD-L1 expression and PD-1 checkpoint pathway, providing a possible direction for studying bacterial carcinogenesis mechanisms. Furthermore, the increased interactions within the microbiome in patients may play roles in the development of CRC. In conclusion, this study identified novel microbiota combinations with discrimination for colorectal tumor, and revealed the potential interactions of gut bacteria with viruses in the adenoma-carcinoma sequence, which implies that the microbiome, but not only bacteria, should be paid more attention in further studies.

## 1. Introduction

Colorectal cancer (CRC) has become the third most common malignancy after breast cancer and lung cancer worldwide [1]. Colorectal adenoma (CRA) is the main precancerous disease of CRC, accounting for 85–90% of all CRC precancerous lesions [2]. As one of the most prominent pathways, sporadic CRC follows the adenoma-carcinoma sequence from normal intestinal mucosa, to progression, to adenoma, and eventually to cancer, which usually takes decades [3,4].

Gut microbial dysbiosis has been demonstrated to play important roles in the occurrence and development of colorectal tumor. Sequencing studies have revealed changes in the gut microbiota of patients with CRC. Compared with healthy controls, the gut microbiota of CRC patients showed a decrease in the abundance of taxa groups, such as *Bifidobacterium* and *Roseburia*, but an increase in the abundance of *Enterococcus*, *Peptostreptococcus*, *Parvimonas*, *Fusobacterium*, and *Porphyromonas* [5,6]. At the species level, the CRC enriched bacterium including *Bacteroides fragilis*, *Escherichia coli*, *Enterococcus faecalis*, *Streptococcus gallolyticus*, *Peptostreptococcus anaerobius*, *Fusobacterium nucleatum*, *Parvimonas micra*, *Porphyromonas asaccharolytica*, and *Prevotella intermedia* acted as promising biomarkers for early detection of intestinal cancer [7]. Recently, the potential of gut microbes as non-invasive diagnostic markers to distinguish patients from healthy individuals has been continuously explored [8,9,10,11]. Dai Z et al.’s study based on four cohorts in multiple countries showed that seven CRC-enriched bacteria could distinguish patients from healthy controls in different populations, with the area under the receiver-operating characteristics curve (AUC) of 0.80 [9]. A retrospective study by Wirbel et al. identified 29 globally representative CRC characteristic gut bacteria, supporting the potential discrimination role of microbiota for CRC [10]. However, the bacterial markers for the CRC precancerous lesions have not been well addressed since most studies only included healthy controls and CRC patients.

In addition to gut bacteria, intestinal viruses may also play roles in identifying patients with CRC [12,13]. A recent study identified five intestinal bacteriophages that were enriched in CRC, and could differentiate CRC from controls [14]. However, existing studies mainly focused on gut bacteria that account for a large proportion of the microbial community; evidence for the roles of viruses in CRC still needs further accumulation. Moreover, the cross-talk between bacteria and viruses in colorectal carcinogenesis remains unclear. Given the potential of gut bacteria and viruses in the early non-invasive detection of CRC, more studies are needed to further explore the impact of enteric microbiota on colorectal neoplasia and identify new diagnostic biomarkers for colorectal tumor.

In this study, metagenomic sequencing of stool samples from healthy controls, CRA, and CRC patients was performed to identify changes in intestinal microorganisms during the development of colorectal tumor, as well as to screen for gut bacterial and viral diagnostic markers that can identify CRA and CRC patients. We also analyzed the association of gut bacteria with viruses to identify cross-kingdom interactions of intestinal microorganisms in patients with CRA and CRC. This study might provide more evidence for the etiology research and early diagnosis of CRC.

## 2. Materials and Methods

### 2.1. Study Population and Design

We recruited pathologically confirmed patients with CRA or CRC, and healthy individuals undergoing physical examination in Wuhan from March 2019 to September 2020. Stool samples were collected from the participants before colonoscopy and frozen in a −80 °C refrigerator within 4 h for long-term storage. The inclusion criteria of participants and measures to prevent contamination during the collection of stool samples are described in Supplementary Methods. A structured questionnaire was used to collect basic information (age, sex, height, weight), personal history of diseases and conditions, family history of cancer, and lifestyle (smoking, alcohol consumption) of the study subjects. The Ethics Committee of Tongji Medical College, Huazhong University of Science and Technology, approved this study protocol. Informed consent was obtained from all patients.

### 2.2. Metagenomic Sequence Data Processing

Fecal shotgun metagenomic sequencing was performed in 35 healthy controls, 29 CRA patients, and 30 CRC patients (Table 1). We used the TIANamp Stool DNA Kit to extract the total DNA from the stool, and performed rigorous quality control of the DNA samples to ensure the usability of the samples and the accuracy of the results of sequencing. The Illumina NovaSeq 6000 sequencing platform was used for sequencing of the qualified metagenomic library. The details of the DNA extraction process, the quality control of the DNA samples, and the subsequent DNA library construction and sequencing process are described in the Supplementary Methods. Next, we performed preprocessing and quality control of the raw sequencing data to obtain high-quality data. The relevant details are described in the Supplementary Methods.

### 2.3. Sequence Taxonomic Annotation

Based on the quality-controlled sequencing data, we filtered out contaminating sequences from humans to perform taxonomic annotation of species with a focus on bacteria. To further annotate the viruses, we filtered out more potentially contaminating sequences, such as bacterial plasmids, complete mitochondrial genomes, etc., to obtain the viral sequences (details in Supplementary Methods). For bacterial-based metagenomic clean reads, MetaPhlAn3 [15] was used to profile the composition of microbial communities from the quality-controlled sequences and determine the microbial composition information of each sample at different taxa levels. The viral reads were assigned to microbial taxa by using the k-mer-based algorithms implemented in Kraken2 taxonomic annotation software [16]. The NCBI nucleotide collection (nt) database, which comprises 174,246 viral taxons (including 3,303,323 viral sequences), was further processed to remove all reverse transcription sequences of RNA viruses (151,088 RNA viral taxons which include 3,036,795 RNA viral sequences) to construct the custom search reference database of Kraken2. Then Bracken software was used to re-estimate the viral taxa abundances based on the Kraken2 results.

### 2.4. Gene Prediction and Metabolic Pathway Functional Annotation

MEGAHIT and Prokka were used to perform gene prediction and quantification of the quality-controlled sequences to obtain the relative abundance of non-redundant genes for each sample. The relevant details were described in the Supplementary Methods. Then the non-redundant genes of the microbiota were mapped to the human-related metabolic pathways in the Kyoto Encyclopedia of Genes and Genomes (KEGG) database to obtain the annotation information of the metabolic functions of the microbiota. The analysis was done with eggNOG mapper-1.0.3 (https://anaconda.org/bioconda/eggnog-mapper).

### 2.5. Microbial Ecological Analysis

We chose the alpha diversity metrics Chao1 index and Shannon index to assess the within-sample diversity. The absolute abundance (operational taxonomic unit (OTU) count number) of species was rarefied by the minimum number of sequences, and then the above indices were calculated. The analysis was done using the R package ‘phyloseq’. The Kruskal–Wallis rank-sum test was used for the multi-group comparison of alpha diversity, and the Wilcoxon rank-sum test was used for the pairwise comparison. We performed the principal coordinate analysis (PCoA) based on Bray–Curtis distances between samples to assess the beta diversity of samples and measure differences in community structure across samples. The results of the PCoA were presented as two-dimensional plots, and the analysis was completed using the R package ‘phyloseq’. In addition, we examined the inter-group differences in community structure by the permutational multivariate analysis of variance (PERMANOVA, i.e., Adonis analysis). The analysis was done using the adonis function (with 999 permutations) in the R package ‘Vegan’.

### 2.6. Difference Analysis

The non-parametric Kruskal–Wallis rank-sum test was used to identify the differences in the relative abundance of bacterial/viral species and metabolic pathways among three groups (control, CRA, and CRC groups). We further performed the Wilcoxon rank-sum test on the species and pathways with significant differences in the Kruskal–Wallis test among the three groups to identify species or pathways that were different in every pairwise comparisons (control vs. CRA, control vs. CRC, and CRA vs. CRC). The *p*-adj value was obtained by Benjamini–Hochberg (BH) correction of the *p* value from the multiple comparisons. A *p* value less than 0.05 was considered statistically significant. For differential species between two groups, species were considered to be enriched in the group with higher mean rank values and depleted in the group with lower mean rank values.

### 2.7. Associations between Species and Pathways

The correlation between species and metabolic pathways was assessed using Spearman’s rank correlation in all samples. The differential species and pathways among three groups (Kruskal–Wallis test results) were selected for the correlation analysis using the R package ‘Hmisc’. The correlation between species and pathways was visualized using heatmap by using the R package ‘pheatmap’.

### 2.8. Correlation Network

The relationships within bacteria, and between bacteria and viruses were estimated using the SparCC algorithm, known for its robustness to microbiome compositional data. The cross-kingdom OTU table (bacterial and viral OTU tables merged) was used as the input file of SparCC [17]. Briefly, the sparse correlations between species were calculated by 20 iterations, and the pseudo *p* values were estimated using 100 bootstrap samplings for each correlation [18]. The analysis was done using the R package ‘SpiecEasi’. The bacteria–bacteria and bacteria–virus associations with two-sided *p* values < 0.05 were shown in the networks, which were mapped using Cytoscape 3.9.1. Next, we evaluated the importance of each node, which represents microbial species, by degree centrality, closeness centrality, and betweenness centrality. Degree centrality indicates the number of nodes directly connected to a specific node, reflecting the number of times the node communicates with other nodes. The closeness centrality emphasizes a node’s value in the interaction network, and a larger metric indicates that the node is more centrally located. Betweenness centrality indicates the potential regulation ability of a node to other nodes. These metrics were calculated using the R package ‘igraph’. We used CHERRY (https://github.com/KennthShang/CHERRY, accessed on 19 August 2022), the latest computational method with the highest accuracy for predicting virus–prokaryotic interactions, to predict bacterial hosts of phages [19]. To improve prediction accuracy, we only show bacterial hosts with prediction scores greater than 0.90.

### 2.9. Microbial Classifier Selection

To determine the diagnostic potential of bacterial and viral taxa in discriminating colorectal disease status, we used a less stringent threshold (*p* value < 0.05) to include more species for model training. A random forest algorithm was used to screen for bacterial/viral diagnostic markers as the microbial classifier based on the relative abundances of species among three groups. Ten-fold cross-validation was performed 10 times to average the error curves, and the value of minimum error in the averaged curve plus one standard deviation at that point was chosen as the cutoff. Then, based on the cutoff value, the microbial feature set with the smallest number of features was screened out as the optimal classifier. The random forest diagnostic model was constructed using the R package ‘randomForest’, and the receiver operating characteristic (ROC) curve of the diagnostic model was plotted using the R package ‘pROC’. The area under the ROC curve (area under curve, AUC), sensitivity and specificity were used to assess the diagnostic performance of the model. 

### 2.10. External Validation Data Acquisition and Analysis

The publicly available human gut microbial metagenomic sequencing datasets were downloaded through the R package ‘curatedMetagenomicData’ [20]. Eleven datasets in this R package contained sequencing data of stool samples from CRC patients and healthy controls. Exclusion of one dataset without deep-sequencing data [12], the remaining 10 metagenomic datasets (1523 samples) from 8 countries [10,21,22,23,24,25,26,27] were included for external validation. The microbial taxonomic composition of all external validation datasets was annotated with MetaPhlAn3 [15]. Meta-analysis was conducted to confirm the individual differential bacterial species identified in our own data (species with a Kruskal–Wallis test *p*-adj value < 0.05 among the three groups) based on external datasets. We used standardized mean difference (SMD) to measure the effect size in the external validation datasets [25]. Heterogeneity of the meta-analysis was assessed using I^2^. A fixed-effect model was used if I^2^ < 0.5; otherwise, a random-effect model was used. Meta-analysis was done using the R package ‘metafor’. The diagnostic models based on gut bacterial markers constructed from the discovery set were validated in the external validation datasets. ROC curves were drawn, and the diagnostic performance of the model was assessed by AUC with the R package ‘pROC’.

## 3. Results

### 3.1. General Characteristics of Intestinal Microorganisms of the Participants

A total of 94 individuals (35 healthy controls, 29 CRA patients, and 30 CRC patients) were recruited from Wuhan, China. The mean age of the study subjects was 57.37 ± 8.32 years. Of the participants, 57 (60.64%) were males and 37 (39.36%) were females. Age, gender, hypertension (HTN), diabetes, body mass index (BMI), smoking, and alcohol consumption were comparable among the three groups (all *p* > 0.05, Table 1). More clinical details on CRA and CRC are shown in Supplementary Table S1. We performed metagenomic sequencing on the fecal samples of the research subjects. A total of 4,002,018,953 raw reads were obtained, with an average of 42,574,669 ± 2,214,780 reads per sample. As a result of the preprocessing and quality control of raw sequencing data, a total of 3,921,818,579 (mean ± sd, 41,721,474 ± 4,658,419) metagenomic clean reads were left for the following bacterial-based taxonomic annotation, and 51,302,062 (mean ± sd, 545,767 ± 1,662,156) viral reads were left for the viral taxonomic annotation. The rarefaction curve reached a plateau, indicating a sufficient sample size to cover the most prevalent microbial genes (Supplementary Figure S1A).

### 3.2. Bacteria Alteration across Groups

**Figure 1:**
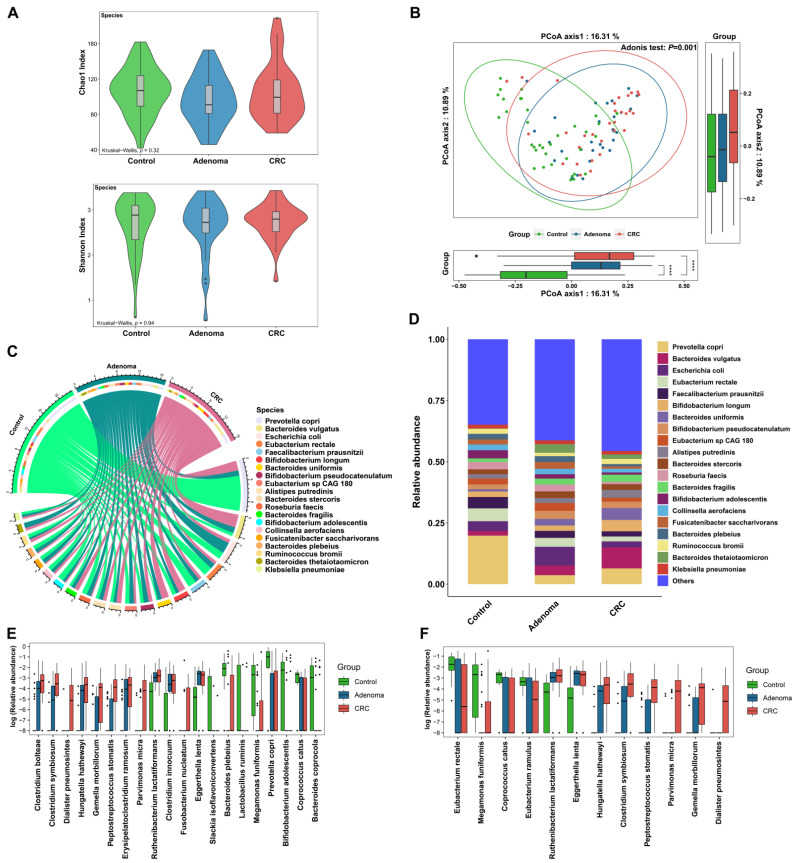
(**A**) The comparison of bacterial alpha diversity among three groups (healthy control (green), CRA (blue), and CRC (red)). (**B**) The comparison of bacterial beta diversity (measured by principal co-ordinates analysis, PCoA) among three groups. (**C**) The chord diagram of sample-species abundance for bacteria. The upper half of the circle represents different groups (control (green), adenoma (blue), and CRC (pink)). The bottom half of the circle shows the top 20 bacterial species in terms of relative abundance, with linked lines showing species–sample associations. Color identification of species is shown in the legend section. (**D**) Bar plots show the abundance distribution of the top 20 bacterial species with the highest overall relative abundance among the three groups. (**E**) The abundance distribution of the top 20 bacterial species with the most significant differences among the three groups (Kruskal–Wallis test, the smaller the *p*-adj value, the more significant the difference). (**F**) The box plots show the relative abundance distribution of the 12 bacterial markers identified by the random forest model among the three groups (the three diagnostic models constructed contain a total of 12 different bacteria, as detailed below and in Figure 2). Black points in subfigures E and F indicate outliers. ****, *p* < 0.0001.

By annotating the clean data with MetaPhlan3 software, a total of 700 microorganisms at the species level were obtained, including 572 bacterial species, 121 viral species, 4 archaeal species, and 3 fungal species. The microbial community was dominated by bacteria, and the overall microbial community structure and distribution of high-abundance species were generally consistent with those of the bacterial community (Figure 1A–D, Supplementary Figure S1B–E, Supplementary Figure S2A–D). Therefore, the results for bacteria were mainly presented. At the species level, no significant difference in the alpha diversity of bacteria was observed among three groups (Figure 1A). PCoA showed the difference in bacterial community structure among the three groups (Figure 1B, adonis *p* = 0.001), which were mainly driven by the difference between control and tumor (CRA and CRC) (control vs. CRA, adonis *p* = 0.001; control vs. CRC, adonis *p* = 0.001), but not between CRA and CRC (adonis *p* = 0.156). In the composition of bacteria, *Prevotella copri* was the species with the highest overall abundance, especially higher in the control group (Figure 1C,D, Supplementary Figure S2C). Among the 572 bacterial species, there were 57 healthy control endemic species, 52 CRC endemic species, and 38 CRA endemic species (Supplementary Figure S2D).

Next, the differences of bacterial species were examined among the three groups. Furthermore, 70 differential bacterial species including *Clostridium symbiosum*, *Fusobacterium nucleatum*, and *Bacteroides fragilis* et al. were identified among the three groups (all *p*-adj < 0.05, Supplementary Tables S2 and S3). *Clostridium Bolteae*, *Hungatella Hathewayi*, *Eggerthella lenta*, and some other bacteria presented consistent changes in CRA and CRC patients compared with controls (Supplementary Table S3). Among the top 20 bacterial species with the most significant differences (according to the rank of *p*-adj values), 12 species were enriched in either the CRA, CRC, or both groups, while 8 species were enriched in the control group (Figure 1E).

### 3.3. Gut Bacteria Distinguish Colorectal Tumor Patients from Controls

**Figure 2:**
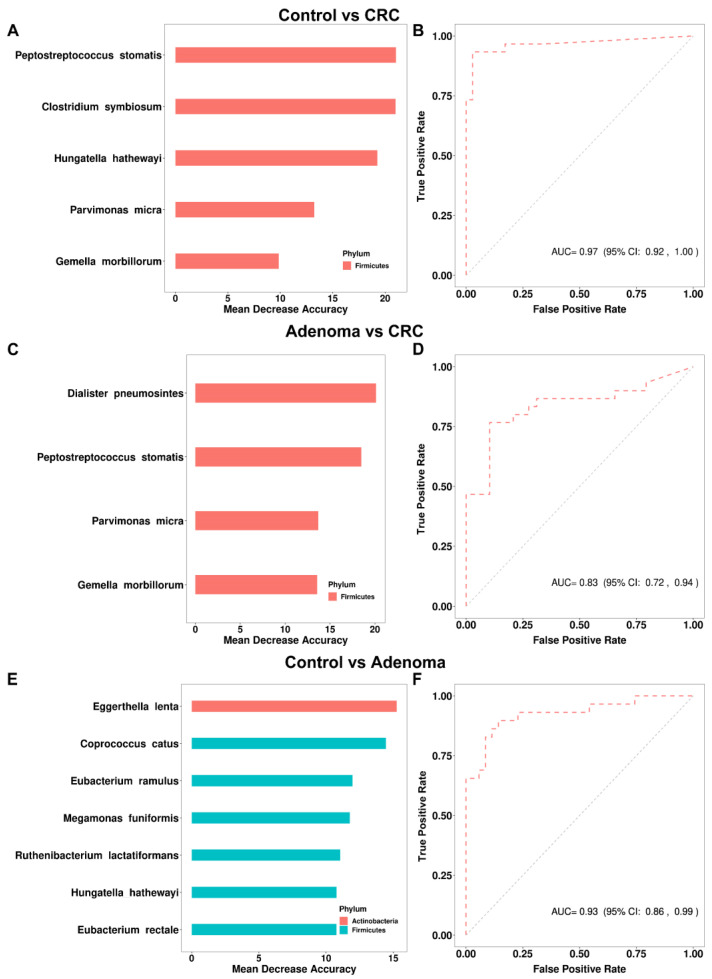
The prediction power of bacterial markers for colorectal adenoma and colorectal cancer. (**A**) Five bacterial markers were identified by the random forest model to discriminate CRC from control. The bar plot shows the average importance score and importance ranking of the five bacteria (measured by mean decrease in accuracy). (**B**) The prediction performance of the bacterial classifier for the classification of CRC and control in our discovery data showed by the area under the curve (AUC) of receiver operating characteristic (ROC). (**C**) Four bacterial markers were identified by the random forest model to discriminate CRC from CRA. The bar plot shows the average importance score and importance ranking of the four bacteria. (**D**) The ROC curve of the classifier for classification of CRC and CRA status in our data. (**E**) Seven bacterial markers were identified by the random forest model to discriminate CRA from control. The bar plot shows the average importance score and importance ranking of the seven bacteria. (**F**) The ROC curve of the classifier for classification of adenoma and control in our data. The legends in Figure 2A,C,E indicate the phylum to which the bacterium belongs.

**Figure 3:**
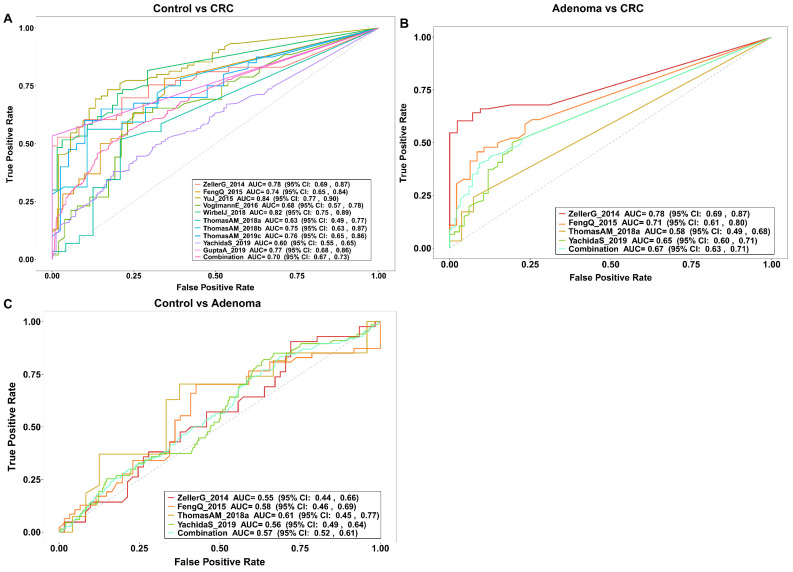
The prediction powers of bacterial classifiers in external validation datasets. (**A**) The prediction performance of the bacterial classifier for the classification of CRC and control status in 10 validation datasets shown by the area under the curve (AUC) of receiver operating characteristic (ROC). (**B**) The ROC curve of the classifier for classification of CRC and CRA status in four validation datasets. (**C**) The ROC curve of the classifier for classification of CRA and control in four validation datasets.

A total of 12 bacteria were identified by the random forest model to discriminate colorectal tumor patients from controls (Figure 1F). We further validated the capacity of the model to discriminate patients from controls in 10 external validation datasets that included either CRA, CRC, or both patients. The general characteristics of the 10 external validation datasets were shown in Table 2. In the discrimination of CRC patients from healthy controls, we identified five bacterial markers (including *Peptostreptococcus stomatis*, *Clostridium symbiosum*, *Hungatella hathewayi*, *Parvimonas micra*, and *Gemella morbillorum*) enriched in CRC in the discovery set as the diagnostic model. The AUC of the model in our training data was 0.97 (95% CI: 0.92–1.00, Figure 2A,B), and the specificity and sensitivity of the model were 0.97 and 0.93, respectively. Except for the *Hungatella hathewayi*, four other bacteria were also significantly enriched in the CRC group in the combined external validation dataset (Supplementary Figure S3A). *Hungatella hathewayi* showed a trend of enrichment in the CRC group in the validation dataset (Supplementary Figure S3B). The AUC of the model was 0.70 (95% CI: 0.67–0.73) based on the combined validation dataset (Figure 3A). In the Chinese validation dataset YuJ_2015, the AUC of the model was up to 0.84 (95% CI: 0.77–0.90). In the discrimination of CRC patients from CRA patients, four bacteria, including *Dialister pneumosintes*, *Peptostreptococcus stomatis*, *Parvimonas micra*, and *Gemella morbillorum*, were identified as the bacterial diagnostic model, with an AUC of 0.83 (95% CI: 0.72–0.94) in the training set, and an overall AUC of 0.67 (95% CI: 0.63–0.71) in the four external validation datasets (Figure 2C,D and Figure 3B). In addition, a model with seven bacteria was constructed to discriminate CRA from controls, with an AUC of 0.93 (95% CI: 0.86–0.99) and 0.57 (95% CI: 0.52–0.61) in the discovery and external validation datasets, respectively (Figure 2E,F and Figure 3C).

### 3.4. Metabolic Pathways Alteration across Groups and Associations between Bacteria and Metabolic Pathways

**Figure 4:**
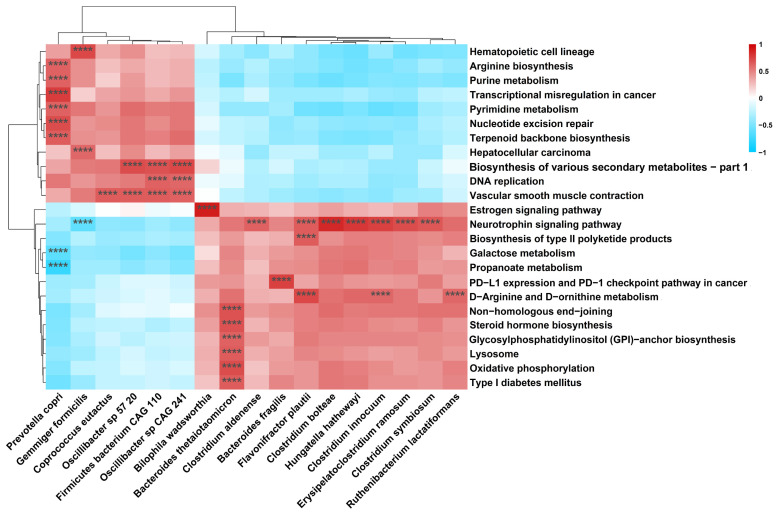
Heatmap of the correlations between bacterial species and metabolic pathways. The heatmap shows the species and metabolic pathways with the absolute values of *Spearman*’s rank correlation coefficient |r_s_| > 0.6 and *p*-adj value < 0.05. ****, *p*-adj value < 0.001.

We assessed the differences in the gut microbial metabolic function among the three groups and identified 64 differential KEGG metabolic pathways (Supplementary Tables S4 and S5). The associations between differential bacterial species and metabolic pathways were explored by Spearman’s rank correlation analysis (Figure 4). *Bacteroides fragilis* presented positive correlation with programmed death-ligand (PD-L1) expression and the programmed death-1 (PD-1) checkpoint pathway in cancer (r = 0.76, *p*-adj = 8.86 × 10^−16^). In addition, *Prevotella copri* had a strong positive correlation with the nucleotide excision repair pathway (r = 0.70, *p*-adj = 9.99 × 10^−13^), and a negative correlation with the propanoate metabolism pathway (r = −0.78, *p*-adj < 3.90 × 10^−20^).

### 3.5. Gut Virome Alteration across Groups

The abundance of viruses were ranked fifth and seventh in the microbial community at the phylum and class level, respectively. The majority of viruses were present in CRC patients (Supplementary Figure S4A,B). Since most of the viruses annotated by MetaPhlan3 software were unclassified, the viruses were further annotated using Kraken2 software which has a better ability to identify viruses. A total of 1875 viral species were annotated by Kraken2. A negative correlation was observed between bacterial and viral community diversities in CRC patients, but not in healthy controls or CRA patients (Supplementary Figure S4C).

**Figure 5:**
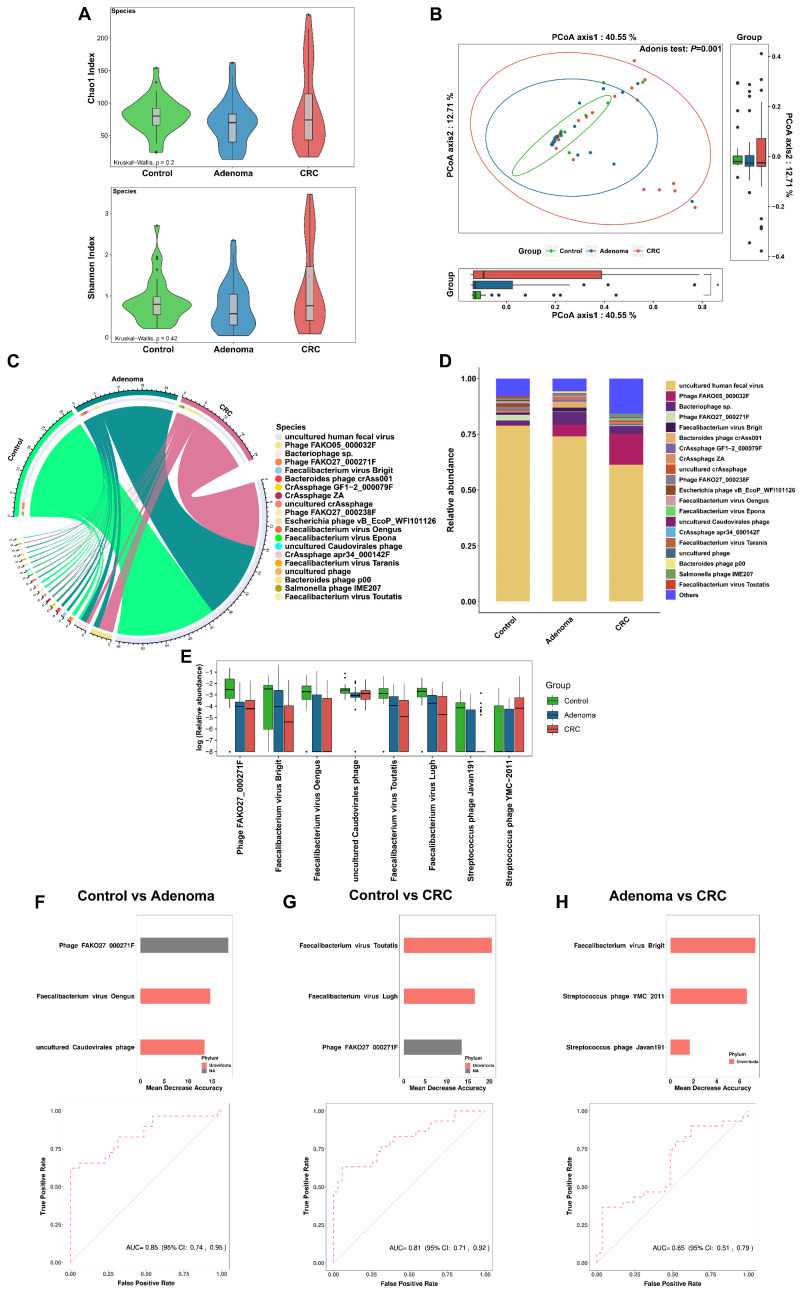
(**A**) The comparison of viral alpha diversity among three groups (healthy control (green), CRA (blue), and CRC (red)). (**B**) The comparison of viral beta diversity (measured by principal co-ordinates analysis, PCoA) among three groups. (**C**) The chord diagram of sample-species abundance for viruses. The upper half of the circle represents different groups (control (green), adenoma (orange), and CRC (pink)). The bottom half of the circle shows the top 20 bacterial species in terms of relative abundance, with linked lines showing species–sample associations. Color identification of species is shown in the legend section. (**D**) Bar plots show the abundance distribution of the top 20 viral species with the highest overall relative abundance among the three groups. (**E**) The abundance distributions of the eight viral markers among the three groups are shown by the box plots. (**F**–**H**) The performance of viral markers for classification of healthy control and disease status in our data. The importance ranking of viral species based on random forest analysis and the prediction performance of the viral markers to discriminate adenoma from control (**F**), CRC from control (**G**), and CRC from adenoma (**H**). The sub-legends in (**F**–**H**) indicate the phylum to which the virus belongs. NA indicates that the virus is not yet classified as a particular phylum in the current classification system (taxonomy browser, NCBI). Black points in subfigures B and E indicate outliers. * *p* < 0.05.

No significant difference was observed in the alpha diversity of gut viruses among the three groups (Figure 5A). PCoA showed the difference in viral community structure (Figure 5B, adonis *p* = 0.001) among the three groups. Among the viruses that could be cultured, *Phage FAKO05_000032F* presented the highest abundance rank (Figure 5C,D). The clustering heatmap showed the distribution of the top 30 viral species in terms of overall abundance among the three groups (Supplementary Figure S4D). There were 351 virus species co-existing among the three groups, of which the highest abundance was the *uncultured human fecal virus* (Supplementary Figure S4E).

The viral difference among the three groups was further explored. The abundance of *Lughvirus* was depleted in CRA and CRC groups at the genus level (Kruskal–Wallis test, *p*-adj < 0.05). Among the three groups, 59 differential viral species were identified. *Phage FAKO27_000271F*, the most significantly different virus species, was depleted in CRA and CRC groups (Supplementary Tables S6 and S7). *Streptococcus phage Javan59*, *Erwinia virus Wellington*, and *Streptococcus phage CHPC663* showed enrichment trends in both CRA and CRC patients compared with controls (Supplementary Table S7).

### 3.6. Gut Virome Distinguishes Colorectal Tumor Patients from Controls

A total of eight viruses were identified by the random forest model to discriminate colorectal tumor patients from controls (Figure 5E). In the discrimination of CRA patients from healthy controls, we identified three viral markers (*Phage FAKO27_000271F*, *Faecalibacterium virus Oengus*, and *uncultured Caudovirales phage*) as the diagnostic model, with an AUC of 0.85 (95% CI: 0.74–0.95) (Figure 4F). The virus markers identified in the discrimination of CRC with controls were *Faecalibacterium virus Toutatis*, *Faecalibacterium virus Lugh*, and *Phage FAKO27_000271F*, with an AUC of 0.81 (95% CI: 0.71–0.92) (Figure 5G). In discrimination of CRC from CRA patients, the markers were *Faecalibacterium virus Brigit*, *Streptococcus phage YMC-2011*, and *Streptococcus phage Javan191*, with an AUC of 0.65 (95% CI: 0.51–0.79) (Figure 5H).

### 3.7. Associations across Microbiome

**Figure 6:**
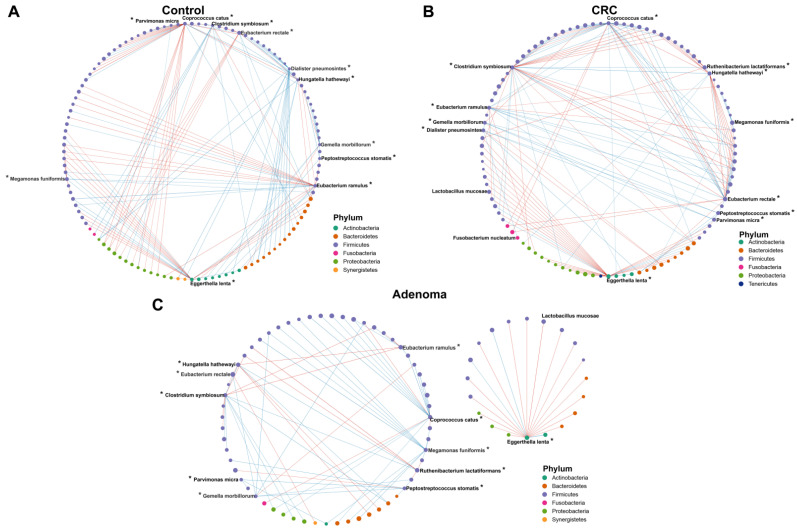
The correlation networks across the bacteria in the control (**A**), CRC (**B**), and CRA (**C**) groups. Lines between dots indicate the significant correlation of species (*p* < 0.05). The blue line indicates a positive correlation, and the red line indicates the presence of a negative correlation. The size of the node is proportional to the relative abundance of species. The nodes are colored according to the phylum to which the species belongs. *, bacterial markers screened by the random forest model.

Correlations within the gut microbiota among the control, CRA, and CRC groups were assessed. We first evaluated the association of 12 bacterial diagnostic markers screened by the random forest model with all bacteria (Figure 6A–C, source data Tables S1–S3). Most of the associations within the bacteria in the healthy controls were broken in the CRA and CRC groups. There were some bacteria-to-bacteria associations in the CRA and CRC groups that were not found in the healthy controls. For example, the positive correlations of *Parvimonas micra* with *Peptostreptococcus stomatis*, *Eggerthella lenta* with *Lactobacillus mucosae*, and *Hungatela hathawayi* with *Ruthenibacterium lactatiformans* were only found in CRA and CRC groups. Then three centrality indexes were used to describe the importance of gut microbiota in the correlation network. Within the gut bacteria, there was no statistical difference in the betweenness centrality among the three groups (Supplementary Figure S5A). The closeness centrality decreased with disease progression (from control to CRC), while degree centrality increased significantly (Supplementary Figure S5B,C).

**Figure 7:**
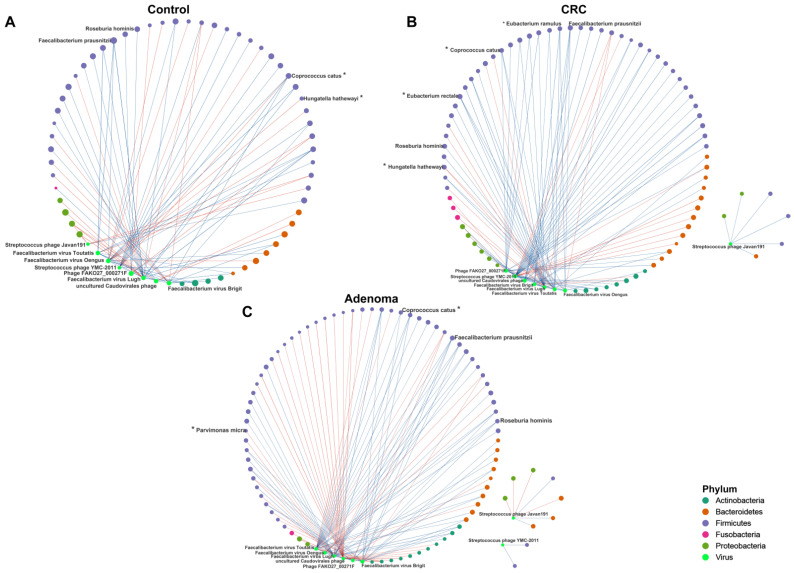
The correlation networks between the viral markers and bacterial species in the control (**A**), CRC (**B**), and CRA (**C**) groups. Lines between dots indicate the significant correlation of species (*p* < 0.05). The blue line indicates a positive correlation, and the red line indicates the presence of a negative correlation. The size of the node is proportional to the relative abundance of species. The nodes are colored according to the phylum to which the species belongs. *, bacterial markers screened by the random forest model. Viruses marked with green node are eight viral markers.

Next, we tested the cross-kingdom associations between the identified viral diagnostic markers and the gut bacteria (Figure 7A,B). Positive correlations between Faecalibacterium virus Toutatis and Coprococcus catus, Faecalibacterium virus Brigit and Faecalibacterium prausnitzii, and Faecalibacterium virus Toutatis and Roseburia hominis existed only within the CRA and CRC groups (Figure 7A,B, source data Tables S4–S6). Centrality analysis also showed that, similar to the correlation within bacteria, the strength of the correlations between viruses and bacteria were significantly higher in the CRC group (Supplementary Figure S5D–F). We further showed the predicted bacterial hosts for phages that differed among the three groups. We observed that *Phage FAKO27_000271F* matched a relatively large number of bacterial hosts and was dominated by *Proteobacteria* and *Firmicutes* bacteria at phylum level. The predicted hosts matched to *Streptococcus phage YMC-2011* were all *Firmicutes* phylum bacteria (Supplementary Figure S6, source data Table S7).

## 4. Discussion

Based on metagenomic sequencing of healthy controls, CRA, and CRC, the current study observed that some bacteria and viruses were enriched in the CRA group and CRC group compared with the control group, which may have impacts on the occurrence and development of colorectal tumors. The gene pathways regulated by aberrant gut microbiome were also identified. The diagnostic model consisting of five bacteria of *Peptostreptococcus stomatis*, *Clostridium symbiosum*, *Hungatella hathewayi*, *Parvimonas micra*, and *Gemella morbillorum* presented verifiable discrimination of CRC patients from healthy controls. Furthermore, the cross-talk across the microbiome in CRA and CRC were identified, which may extend the understanding of dysbiosis in colorectal tumorigenesis.

CRC-related bacteria were identified in the current study, such as *Peptostreptococcus stomatis*, *Clostridium symbiosum*, *Hungatella hathewayi*, *Eggerthella lenta*, *Bacteroides fragilis*, *Gemella morbillorum*, *Parvimonas micra*, *Fusobacterium nucleatum*, etc. (Supplementary Tables S2 and S3), which were consistent with previous studies [10,24,25,26,28,29,30]. Besides, we also identified the enrichment of *Blautia hansenii*, *Streptococcus sanguinis*, *Enterococcus faecalis*, and *Oxalobacter formigenes* in the CRA group, and the same changing trend of these bacteria was observed in existing studies [21,22,25,26]. Since metagenomic studies on the association between gut bacteria and CRA are still limited and the population heterogeneity is large, the general characteristics of gut bacteria in CRA need more exploration. Interestingly, we found that some species (such as *Clostridium symbiosum*, *Clostridium bolteae*, *Hungatella hathewayi*, *Eggerthella lenta*, and *Bacteroides fragilis* et al.) presented consistent changes in CRA and CRC patients compared with controls. Supportingly, previous studies have also shown similar changes of these bacteria in CRA and CRC patients [31,32,33,34], implying that some gut bacteria may play a role throughout colorectal carcinogenesis. Although functional studies have revealed part of the carcinogenic mechanisms of these bacteria, including damaging the intestinal barrier, activating the oncogenic signaling pathway, promoting proliferation of the colon, and suppressing the apoptosis of cancer cells [32,35], our understanding of the carcinogenesis roles of bacteria is still limited. More studies are still needed to draw a comprehensive profile of the carcinogenic mechanisms of tumor-related bacteria.

In the discrimination of CRC patients from controls, our study constructed a diagnostic model consisting of five gut bacteria (Figure 2A,B). The diagnostic model demonstrated verifiable effect in the external validation datasets involving 10 cohorts from multiple countries, with an AUC of 0.70 (95% CI: 0.67–0.73) for the combined population, and a maximum AUC of 0.84 (95% CI: 0.77–0.90) in a Chinese cohort (Figure 3A). In Wirbel et al.’s meta-analysis of the metagenomic sequencing results of populations from seven different countries, 29 core species significantly enriched in CRC were identified, revealing the universal characteristics of CRC patients worldwide. They also demonstrated that the trained CRC gut microbiota classifier from a single study maintained accuracy in other studies, with the average AUC of the diagnostic models varying from 0.65 to 0.81, similar to our findings [10]. In the discrimination of CRA patients from controls, the diagnostic model based on seven bacteria in the current study achieved an AUC of 0.57 (95% CI: 0.52–0.61) in the combined data of four external validation datasets (Figure 3C). The limited discrimination of gut bacteria for CRA was also founded in a recent meta-analysis which showed a maximum AUC of 0.58 when validating diagnostic models derived from individual cohorts [25]. Consistent with the previous view that adenoma presented a similar microbiome with healthy controls [21,22], these results suggest that microbiological features only possess partial prediction to CRA. Overall, the above results implied that gut microbiota might possess a good discrimination for CRC, but limited discrimination for CRA. However, more evidence from further studies is still needed.

Besides the dysbiosis of gut bacteria, we also identified maladjustment of viruses in the development of colorectal tumor, including *Streptococcus phage YMC-2011*, some *Streptococcus phages* and some *Streptococcus satellite phages* enriched in CRC. In addition, *Streptococcus phage Javan59*, *Erwinia virus Wellington*, and *Streptococcus phage CHPC663* showed increasing trends in both CRA and CRC groups (Supplementary Tables S6 and S7). In our data, the AUC of the viral markers to differentiate CRC from controls was 0.81 (95% CI: 0.71–0.92) (Figure 5G). The studies on viral markers for CRC are limited. Moreover, the discrimination of virial markers varied across studies, with AUC ranging from 0.51 to 0.80 [13,36]. These results imply the potential of gut virome in discrimination of CRC from controls, although not as prominent as gut bacteria. However, the similarity of gut viral markers for colorectal tumor across studies is limited, which might be due to the high specificity of individual enterovirome, ongoing discovery of new viruses, and shortage of viral reference genome accurate to the species level [37,38]. In the latest research by Zhao et al. [39], the viral genome has been expanded using ultra-deep metagenomic sequencing. Although this new identification scheme for gut virome has high requirements on sequencing costs and computing resources, it provides a new method for deeper understanding of gut viruses in colorectal tumorigenesis.

In the co-abundance analysis of the gut microbiome, we observed more bacterial intra-kingdom associations in patients with CRA and CRC compared to controls, manifested by decreases in closeness centrality and increasing trends in degree centrality in CRA and CRC groups. Similarly, more interactions between viruses-bacteria were observed in the CRC group. This is consistent with a recent study that observed increased gut microbiota intra- and inter-kingdom interactions in CRC [40], which implies that the complex microbial dysbiosis may play roles in colorectal carcinogenesis. In the host prediction of phage, we observed that *Streptococcus phage YMC-2011* and its predicted host *Streptococcus thermophilus* both showed a tendency to be enriched in the CRC group. Focusing on this interaction between phages and their hosts may help to further understand the dynamic relationship between gut microbes in patients with CRC.

In the analysis of associations between bacterial species and differential metabolic pathways, we found that *Prevotella copri*, a deleted species in tumor patients, was positively correlated with the nucleotide excision repair pathway, but negatively related with the propanoate metabolism pathway. The nucleotide excision repair pathway is important for the repair of DNA damage caused by carcinogens, ionizing radiation, and ultraviolet radiation [41]. It has been shown that the microbiota can promote tumorigenesis in DNA mismatch repair-deficient CRC models. The failure to repair damaged DNA has the potential to lead to CRC [42]. The positive association between *Prevotella copri* and the nucleotide excision repair pathway suggests that *Prevotella Copri* may inhibit the occurrence and development of colorectal tumor through promoting the repair of DNA damage. Propionic acid is a common short-chain fatty acid. Short-chain fatty acids could inhibit the proliferation and differentiation of colon cancer cells [43]. The up-regulation of the propanoate metabolism pathway may cause decreased propionic acid, and therefore weakened tumor-suppressive effect. Nguyen et al. showed that fecal propionic acid content could be predicted by *Prevotella copri*, suggesting that *Prevotella copri* may have a regulatory role in metabolism of propionic acid [44]. In addition, we found a positive correlation between *Bacteroides fragilis* and PD-L1 expression and the PD-1 checkpoint pathway. *Bacteroides fragilis* was the 21st ranked bacterium with regard to difference (Supplementary Table S2, ranked by *p*-adj value). The binding of PD-L1 on cancer cells to PD-1 on the surface of tumor-infiltrating T cells could inhibit the recognizing and killing effects of T cells towards cancer cells [45]. The activation of PD-L1 expression and the PD-1 checkpoint pathway has been demonstrated to be associated with poor prognosis of CRC, and inhibition of PD-L1 signaling could improve the prognosis of CRC [45]. Immune checkpoint blockade (ICB) is commonly used in cancer immunotherapy. Some bacteria have been demonstrated to modulate the anticancer effects of ICBs, including PD-1 and PD-L1 inhibitors [46]. The positive correlation between *Bacteroides fragilis* and PD-1 checkpoint pathway implies that *Bacteroides fragilis* may play an oncogenic role by inhibiting anti-tumor immunity, and may increase tumor neoantigens by up-regulating PD-L1 expression, in order to to improve the tumor response to PD-1/ PD-L1 blockade therapy. Although there is no direct evidence on how *Bacteroides fragilis* improves immunotherapy through the PD-L1 expression and PD-1 checkpoint pathway thus far, the study by Vetizou et al. revealed that Bacteroides fragilis could improve the therapeutic effect of another immune checkpoint inhibitor CTLA-4 blockade on melanoma mice, which to some extent supports our inference [47]. However, further experimental evidence is still needed.

The current study has several strengths. First, the study identified the combination of five gut bacteria that can be used to identify patients with CRC, which to our knowledge contains the lowest number of features and can be validated in multiple cohorts for the gut microbiota diagnostic model. These five bacteria can be detected in almost all published metagenomic datasets and showed correlations with CRC [10], which is expected to become a non-invasive diagnostic marker for CRC. Second, the study revealed colorectal tumor-related viruses, and proposed the potential of viruses to discriminate patients with CRC. Third, the study revealed the potential interactions of gut bacteria with viruses and within bacteria in the adenoma-carcinoma sequence, which may extend our understanding on dysbiosis in colorectal carcinogenesis. There are also some limitations. First, the sample size of our study is limited. To improve the validity of our findings, the publicly available metagenomic data of colorectal tumor were downloaded as external validation datasets. As a case-control study design, a limitation of causal inference exists. We can only infer that certain microorganisms might be potential risk factors for colorectal neoplasms. Second, although strict inclusion and exclusion criteria were implemented at the sample collection stage to reduce bias, detailed lifestyle factors were not collected. However, it is possible that lifestyle influences the occurrence and development of colorectal tumors through gut microbiota, and whether the lifestyle acts as a confounder needs further exploration. Third, the external metagenomic sequencing was annotated by MetaPhlan3 software, which has limited ability to annotate the virome, thus the external validation of viral markers was not conducted. We also did not extract viral RNA from feces in this study, which might cause inadequate annotation for viruses, especially for RNA viruses. In addition, although we have used the best predictive software currently available, the known virus–host interactions are still lacking, and not all viruses share common regions with their host genomes, thus the hosts of some phages failed to predict. Finally, the diagnostic model of adenoma versus control presented limited test efficacy in external validation datasets, which implied the possible overfitting of the training model. Besides, this might be attributed to the small number of adenoma-related datasets included, and the regional and ethnic differences in gut microbes.

## 5. Conclusions

In conclusion, this study identifies the gut microbial dysbiosis in CRA and CRC, expanding the biomarkers that can be used to diagnose CRA and CRC. Functional pathways that may play roles in the regulation of colorectal tumor by gut bacteria were identified, providing some possible directions for further mechanism research. In addition, some unique microbiome cross-talk identified in CRA and CRC may extend the understanding of gut dysbiosis in the development of colorectal tumor.

## Figures and Tables

**Table 1:** Characteristics of colorectal adenoma cases, colorectal cancer cases, and controls in our discovery data.

Characteristics	Control	Adenoma	CRC	Statistics *	*p* Value
	(*n* = 35)	(*n* = 29)	(*n* = 30)		
Age ($\bar{x}$ ± sd)	57.53 ± 7.70	56.71 ± 8.21	57.82 ± 9.30	0.14	0.870
Gender, *n* (%)				0.69	0.710
male	20 (57.14)	17 (58.62)	20 (66.67)		
female	15 (42.86)	12 (41.38)	10 (33.33)		
Hypertension, *n* (%)				2.61	0.271
without HTN	27 (77.14)	19 (65.52)	25 (83.33)		
with HTN	8 (22.86)	10 (34.48)	5 (16.67)		
Diabetes, *n* (%)					
without DM	31 (88.57)	25 (86.21)	25 (83.33)	0.06	0.930
with DM	4 (11.43)	4 (13.79)	5 (16.67)		
BMI ($\bar{x}$ ± sd)	23.09 ± 2.39	24.01 ± 2.60	22.51 ± 2.38	2.82	0.065
Smoking, *n* (%)				0.16	0.923
smoking now	10 (28.57)	7 (24.14)	8 (26.67)		
no smoking now	25 (71.43)	22 (75.86)	22 (73.33)		
Drinking, *n* (%)				0.19	0.907
drinking now	6 (17.14)	6 (20.69)	5 (16.67)		
no drinking now	29 (82.86)	23 (79.31)	25 (83.33)		

* For quantitative variables, the mean and standard deviation are shown. Differences among groups are compared with one-way analysis of variance (ANOVA). The statistics are F values. Categorical variables are described by frequency (percentages), and group comparisons are made by chi-square test/Fisher exact probability test with the statistic of chi-square value/table probability (P).

**Table 2:** General characteristics of the external validation datasets.

Dataset	Group (N)		Gender_Male (%)	Age $\bar{x}$ ± sd	Country
ZellerG_2014	Control	61	45.90	60.57 ± 11.39	FRA
	Adenoma	42	71.43	62.95 ± 9.11	
	CRC	53	54.72	66.81 ± 10.88	
FengQ_2015	Control	61	59.02	66.97 ± 6.45	AUT
	Adenoma	47	48.94	66.49 ± 7.86	
	CRC	46	60.87	67.07 ± 10.91	
ThomasAM_2018a	Control	24	54.17	67.92 ± 7.01	ITA
	Adenoma	27	59.26	62.89 ± 8.67	
	CRC	29	79.31	71.45 ± 8.23	
YachidaS_2019	Control	251	54.18	60.81 ± 12.64	JPN
	Adenoma	67	71.64	63.15 ± 9.12	
	CRC	258	62.40	62.72 ± 9.64	
YuJ_2015	Control	53	62.26	61.83 ± 5.70	CHN
	CRC	75	64.00	65.93 ± 10.57	
VogtmannE_2016	Control	52	71.15	61.23 ± 11.03	USA
	CRC	52	71.15	61.85 ± 13.58	
WirbelJ_2018	Control	65	56.92	55.97 ± 12.15	DEU
	CRC	60	60.00	63.45 ± 12.64	
ThomasAM_2018b	Control	28	57.14	57.81 ± 8.26	ITA
	CRC	32	71.88	58.44 ± 8.39	
ThomasAM_2019c	Control	40	60.00	63.23 ± 12.17	JPN
	CRC	40	52.50	59.05 ± 12.83	
GuptaA_2019	Control	30	40.00	41.50 ± 16.90	IND
	CRC	30	60.00	59.80 ± 7.81	

## Data Availability

The datasets presented in this study can be obtained by contacting the corresponding author.

## Supplementary Materials

The following supporting information can be downloaded at: https://www.mdpi.com/article/10.3390/cancers14194662/s1, Reference [48] is cited in the Supplementary Methods. Supplementary Materials including Supplementary Methods, Supplementary Figures S1–S6, Supplementary Tables S1–S7, and Source Data Tables S1–S7.
