# Supplementary material for: Expansion of Colorectal Cancer Biomarkers Based on Gut Bacteria and Viruses
Source: Cancers (Basel). 2022 Sep 25;14(19):4662. doi: 10.3390/cancers14194662 (PMC9563090; doi:10.3390/cancers14194662)
Supplement: Supplementary file 1 [file cancers-14-04662-s001.zip › Supplementary Figures S1¿CS6.pdf]

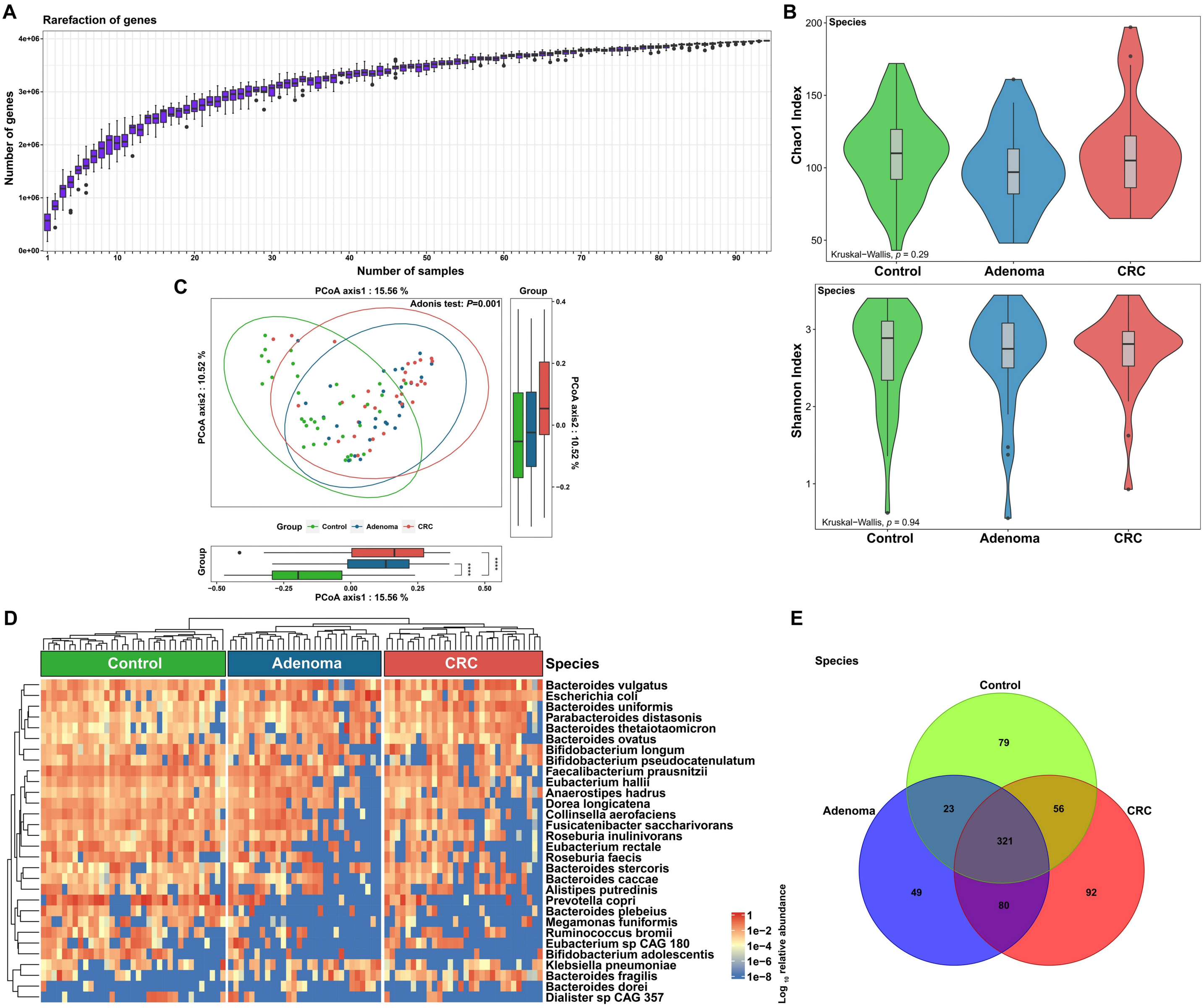

Supplementary Figure S1. The alteration of the gut microbiome among three groups. (A) Sample-based rarefaction curve shows the cumulative number of sequenced genes with increasing sample size. The horizontal axis shows the number of randomly selected samples from all samples, and the vertical axis indicates the corresponding total number of genes. (B) The comparison of microbial alpha diversity among three groups (healthy control (green), CRA (blue), and CRC (red)). (C) The comparison of microbial beta diversity among three groups. (D) Heatmap shows the distribution of the top 30 species in overall abundance (gut microbiome abundance, including bacteria, viruses, and fungi) among the three groups. (E) The number of common and unique taxa among the three groups of microbial species (including bacteria, viruses, and fungi).

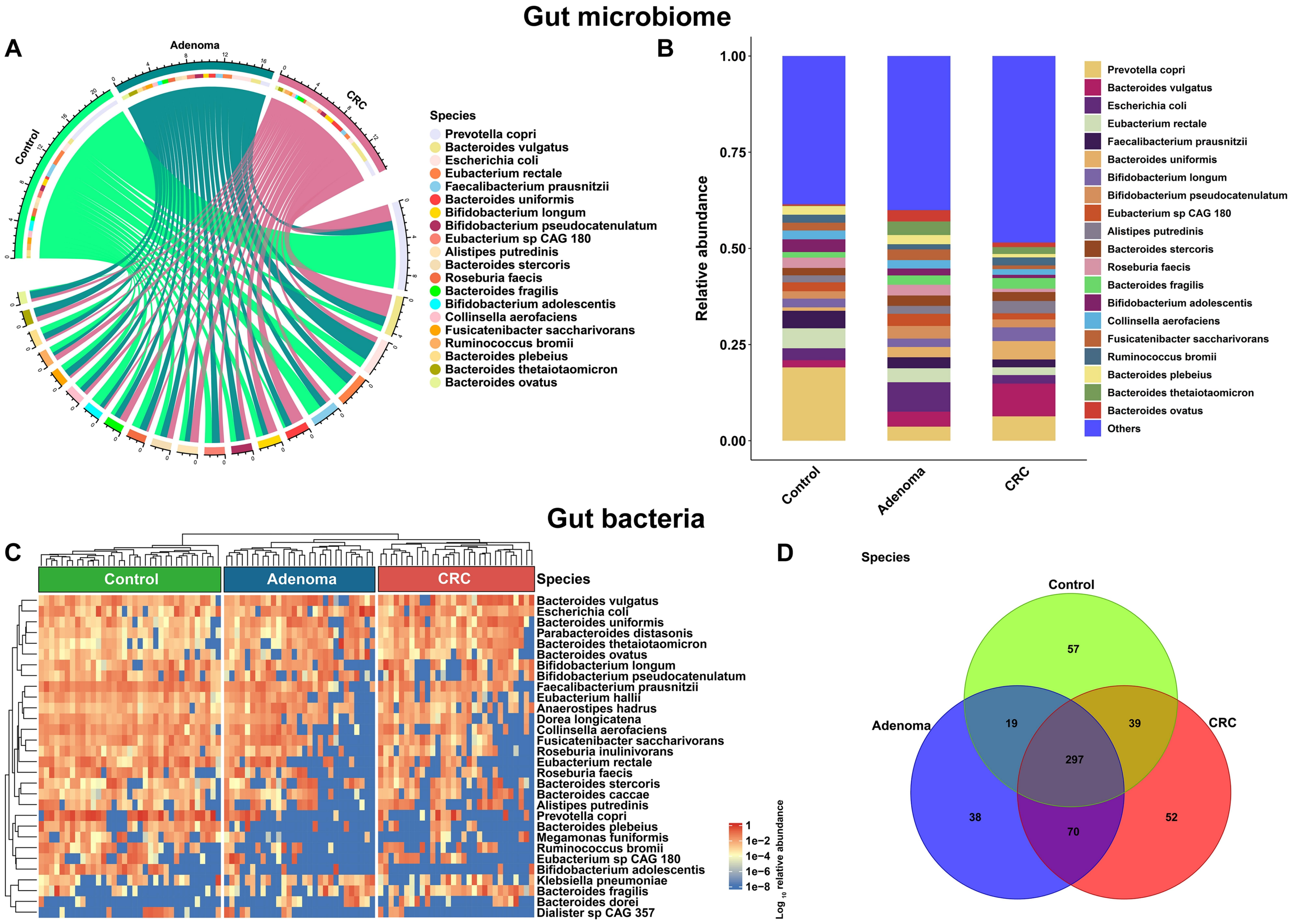

Supplementary Figure S2. (A) The chord diagram of sample-species abundance for microbiome. The bottom half of the circle shows the microbial taxa in terms of relative abundance, with linked lines showing species-sample associations. Color identification of species is shown in the legend section. The upper half of the circle represents different groups (control (green), adenoma (blue), and CRC (pink)). (B) Bar plots show the abundance distribution of the top 20 microbial species with the highest overall relative abundance among the three groups. (C) Heatmap shows the distribution of the top 30 species in overall relative abundance (gut bacterial abundance) among the three groups. (D) The number of common and unique taxa among the three groups of bacterial species.

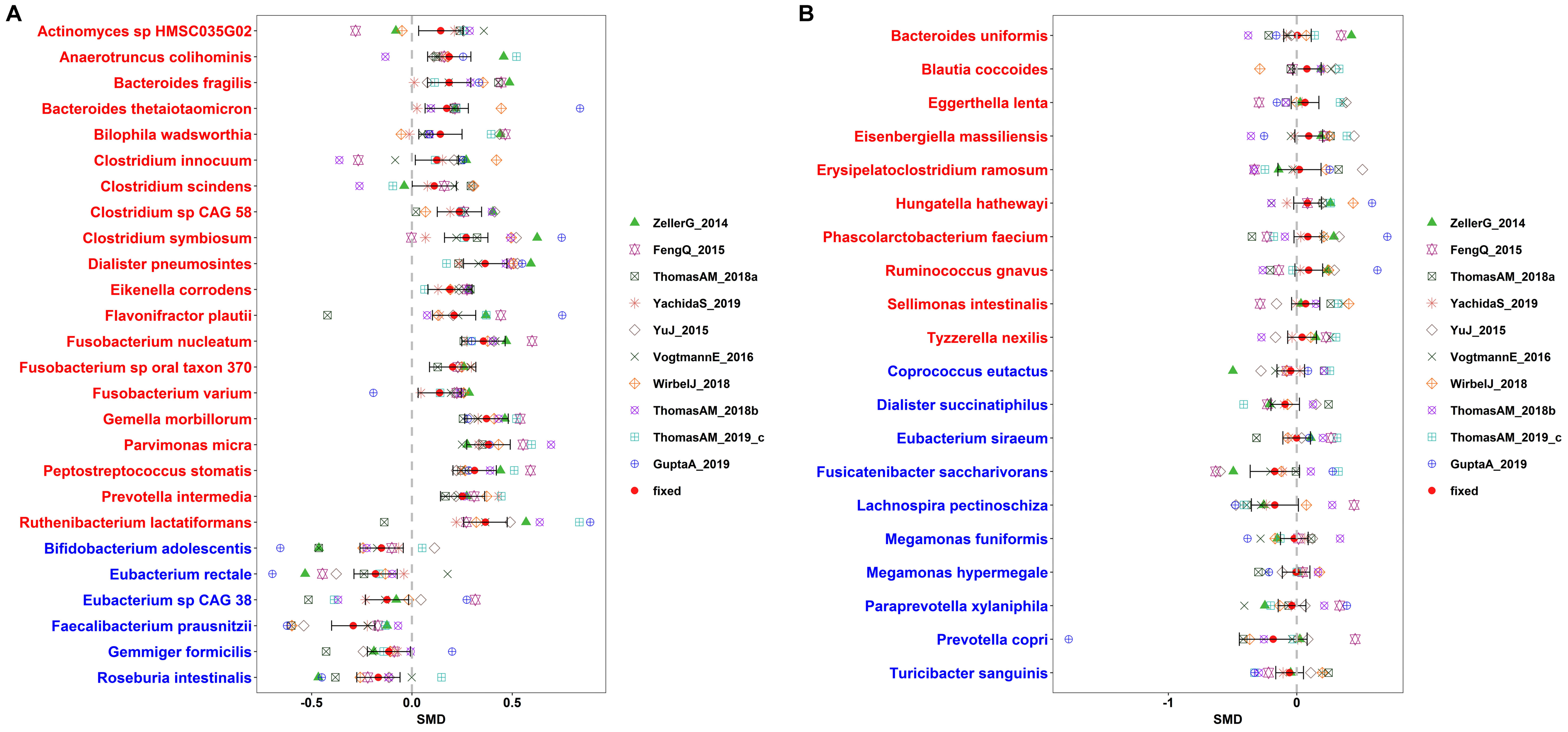

Supplementary Figure S3. External validation of differential bacterial species (Species with *P*-adj values less than 0.05 for the Wilcoxon rank-sum test between the healthy control and CRC group. *P*-adj value was obtained by Benjamini-Hochberg (BH) correction of the *P*-value). (A) In comparison between colorectal cancer and healthy control in the validation set, the direction of the difference for these species was consistent with that in the discovery set, and the difference was statistically significant. (B) In comparison between colorectal cancer and healthy control in the validation set, the direction of the difference for these species was consistent with that in the discovery set. However, the differences were not statistically significant in the external validation set. The Y-axis indicates the different species. The red text indicates that the species is enriched in the CRC group, while the blue indicates that the species is enriched in the healthy control group. SMD, standardized mean difference. Pooled, combined effect values (from meta-analysis) for the ten validation data sets.

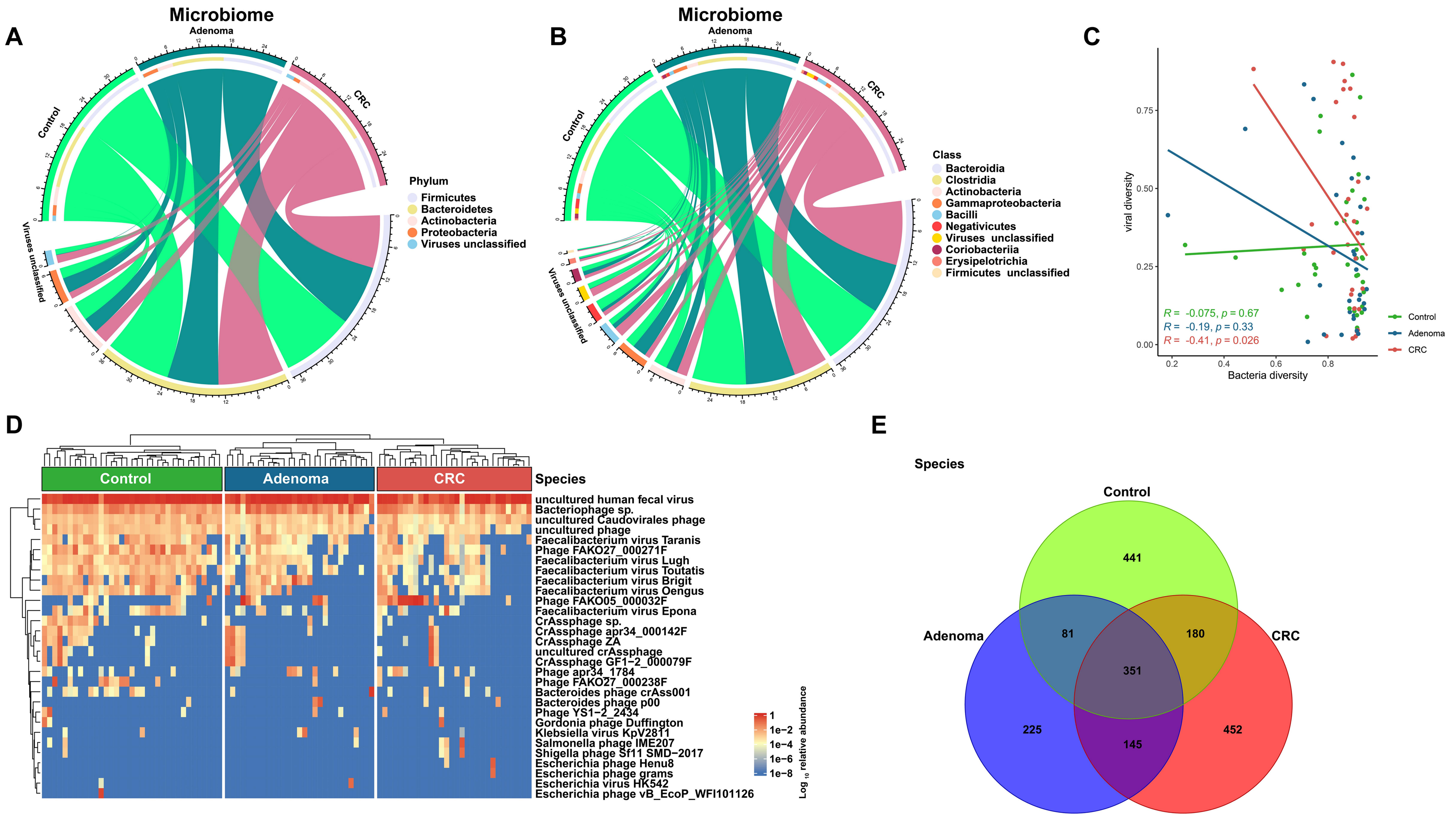

Supplementary Figure S4. (A-B) The chord diagram of sample-species abundance for microbiome at phylum (A) and class (B) levels. The bottom half of the circle shows the microbial taxa in terms of relative abundance, with linked lines showing phylum/class-sample associations. Color identification of phylum/class is shown in the legend section. The upper half of the circle represents different groups (control (green), adenoma (blue), and CRC (pink)). (C) Association between bacterial species diversity and viral species diversity (Simpson index, Spearman rank-order correlation). (D) Heatmap shows the distribution of the top 30 species in overall abundance (gut viral abundance) among the three groups. (E) The number of common and unique taxa among the three groups of viral species.

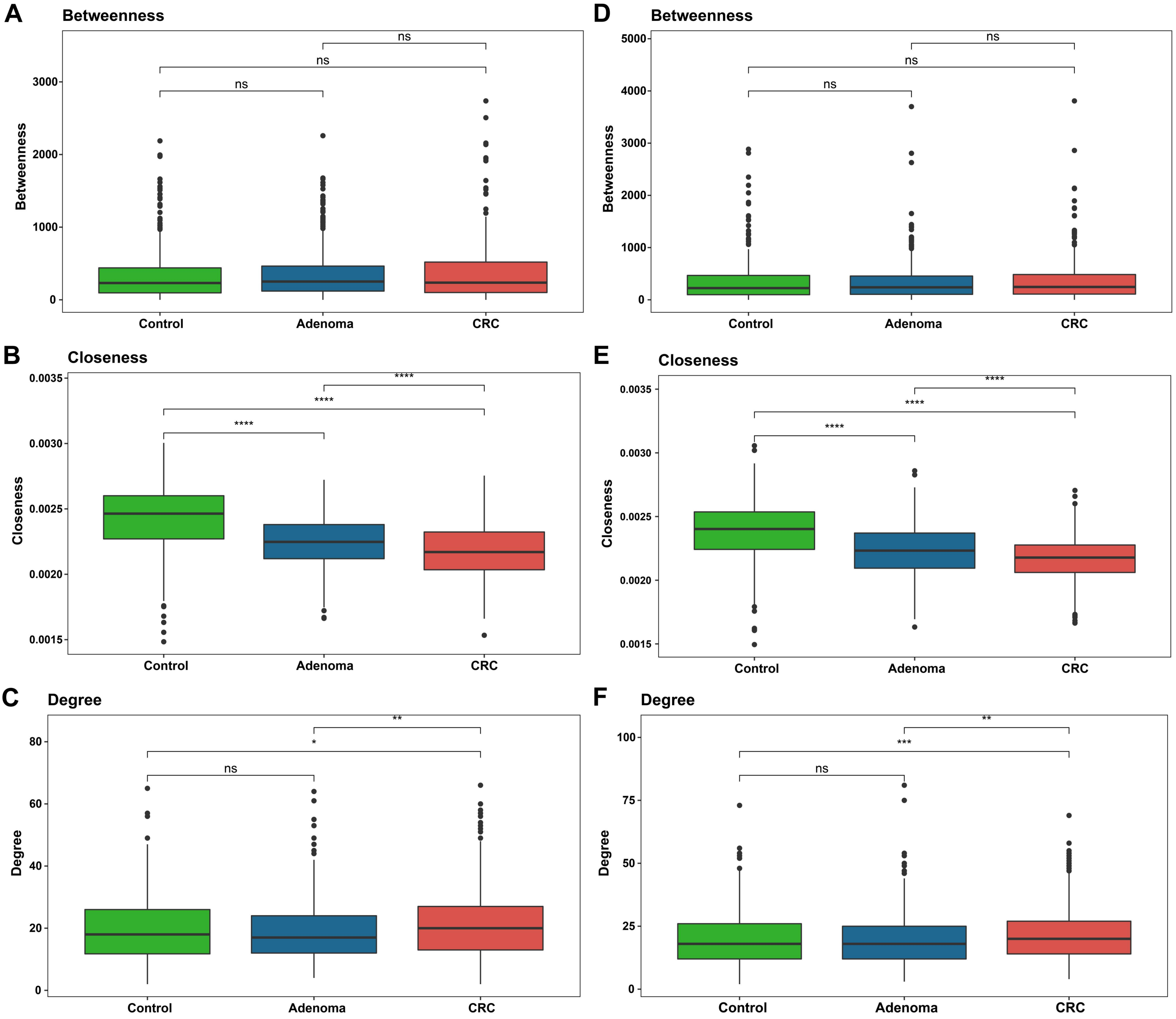

Supplementary Figure S5. The centrality indexes alteration in adenoma and CRC. (A-C) The betweenness centrality (A), closeness centrality (B), and degree centrality (C) of node's importance within the co-abundance correlation of bacteria among the control (green), adenoma (blue), and CRC (red) groups. (D-F) The betweenness centrality (D), closeness centrality (E), and degree centrality (F) of node's importance between the co-abundance correlation of viral markers and bacteria among the three groups. *, $P < 0.05$; **, $P < 0.01$; ****, $P < 0.0001$; ns, no statistical difference.

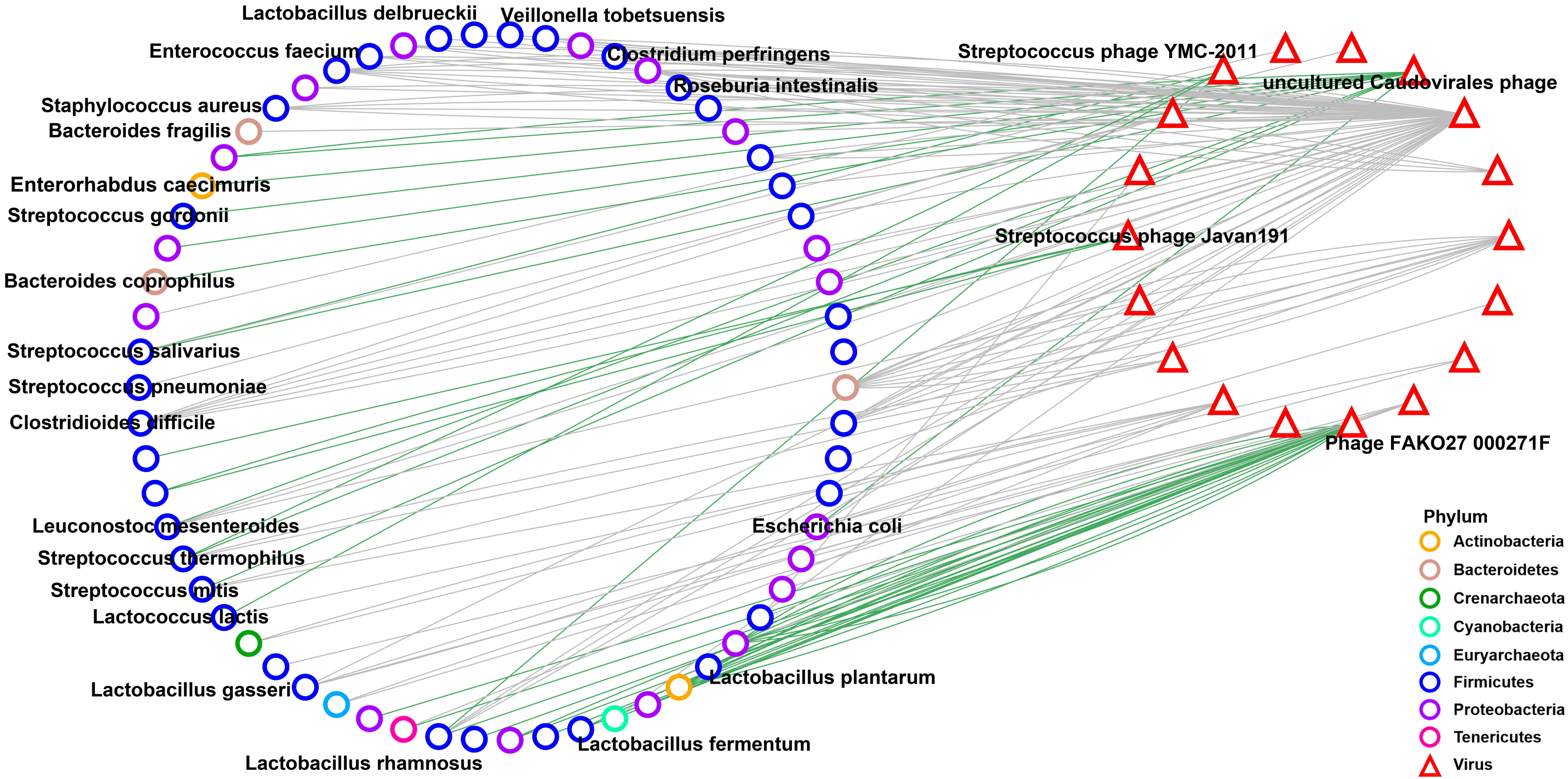

Supplementary Figure S6. Prediction of prokaryotic hosts for viruses (mainly phages). Red triangles represent viruses, and different colored circles represent different predicted bacterial hosts. The phyla corresponding to bacterial hosts are represented by different colors, and the color-phylum correspondence is shown in the legend. Viruses and their corresponding hosts are connected by straight lines, and the viral markers identified in this study are connected with their corresponding hosts by green straight lines.
