# Supplementary material for: Expansion of Colorectal Cancer Biomarkers Based on Gut Bacteria and Viruses
Source: Cancers (Basel). 2022 Sep 25;14(19):4662. doi: 10.3390/cancers14194662 (PMC9563090; doi:10.3390/cancers14194662)
Supplement: Supplementary file 1 [file cancers-14-04662-s001.zip › Supplementary methods.pdf]

Supplementary methods

Sample collection

We recruited healthy controls, patients with colorectal adenoma and colorectal cancer from March 2019 to September 2020, and collected stool samples from the study participants. The inclusion criteria include pathologically confirmed CRA or CRC patients, without histories of tumor or inflammatory bowel disease. Those who were not primary tumors, had undergone radiotherapy or chemotherapy, had undergone gastrointestinal surgery within three months, had used antibiotics or probiotic preparations within three months, or had received colonoscopy within one month were excluded. We instructed the participants to collect approximately 5 g of fresh stool on their own using a sterile stool specimen cup. During the collection process, blood and urine contamination should be avoided, and samples should be taken from the middle and posterior segments as close to the interior of the stool as possible.

DNA extraction of stool samples

The DNA extraction procedure for stool samples was strictly in accordance with the protocol of the TIANamp Stool DNA Kit[1]. The DNA extracts were stored in a -80 ℃ refrigerator for subsequent detection. Afterwards, the extracted DNA was subjected to quality control with the following main processes: 1) The NanoDrop 2000 Spektralphotometer (Thermo Fisher, USA) was used to detect DNA purity and to perform preliminary quantification of DNA concentration. OD260/280 in the range of 1.8-2.0 was considered a qualified sample. 2) The DNA integrity was examined by agarose gel electrophoresis. Samples with DNA bands concentrated above 500bp and no obvious concentrated bands below 500bp were considered qualified samples. 3) The Qubit 4 Fluorometer (Thermo Fisher, USA) was used for accurate quantification of DNA concentrations. DNA concentrations greater than 50 ng/ul were considered qualified samples.

DNA library construction, quality control and sequencing

The Bioruptor sonication system was used to randomly interrupt the qualified DNA samples. Then we selected fragments with appropriate length and used DNA Sequencing Kit (NEXTflex, US) to complete the metagenomic library construction through the flow of DNA end repair and adenylation, adapter ligation, PCR amplification, and bead cleanup steps. Then quality control was carried out for the constructed library by the following steps: 1) Qubit 4 Fluorometer was used for initial quantification of the library and diluted to 2ng/ul. 2) The insert size of the library was detected using the 2100 Bioanalyzer Instrument (Agilent, US), and the insert size of each sample was 350bp. 3) The ABI Veriti-Well 384 Thermal Cycler (Applied Biosystems, US) and HT DNA 1K Reagent Kit (LabChip, US) were used to amplify the library and quantify the effective concentration accurately. The effective concentration of the library should be greater than 3 nM. Illumina NovaSeq 6000 sequencing platform and NovaSeq 6000 S4 Reagent Kit (Illumina, US) were used for sequencing of the qualified metagenomic library. The sequencing platform read the fluorescence signal by laser scanning and then analyzed it to determine the DNA sequence information.

Pre-processing and quality control of raw sequencing data

For the raw reads after sequencing, Fastp (version 0.20.0) software was used to remove low-quality data, including primer sequences, adapter sequences, reads with average quality of less than 20, reads with a base quality of one window less than 15 based on a 4-base sliding window algorithm, reads with more than 5 N bases (base pair not identified) or reads with lengths less than 50bp. Next, for bacterial-based analysis, the software Bowtie2 (version 2-2.1.0) with 'very sensitive' default settings was used to align with the human reference genome hg38 sequence and filter out contaminating sequences derived from humans.

In our results, higher virus abundance rankings were observed at the phylum level and

the class level (see Results section in the main text). We further preprocessed the sequences for virus annotation following the method provided by Geicho et al[2]. Briefly, we aligned, identified, and discarded potentially contaminating sequences. For the viral-based analysis, reads were mapped against an indexed database of 9 mammalian genomes (hg38, felCat8, rn6, canFam3, mm10, susScr3, galGal4, rheMac8 and bosTau8, accessed on March, 2021, University of California Santa Cruz (UCSC) Genome Browser), 33,395 complete bacterial plasmids (National Center for Biotechnology Information (NCBI) RefSeq database, accessed on March, 2021), 11,345 complete mitochondrial genomes (NCBI-RefSeq database, accessed on March, 2021), 6,093 UniVec sequences (NCBI-RefSeq database, accessed on March, 2021), using the same default settings. Reads that could be mapped to the indexed database were considered as potential sources of human habitat/laboratory-associated or extrachromosomal sequence contaminants , and these reads were discarded.

Gene prediction of quality-controlled sequences

The quality-controlled sequences were assembled into contigs using MEGAHIT. Prokka was used to predict the assembled contigs (not less than 300bp in length) for Open Reading Frame (ORF) prediction. CD-HIT was used to cluster the predicted gene sequences (parameters: identity ⩾ 95%, coverage ⩾ 90%), and the obtained representative sequences were regarded as the gene catalogue (non-redundant gene set). Relative abundance information of non-redundant genes in each sample was calculated using Salmon.

Supplementary reference

1. TIANGEN. TIANamp Stool DNA Kit: Rapid extraction of high quality genomic DNA from various stool samples [Available from: https://en.tiangen.com/content/details_43_4245.html.

2. Nakatsu G, Zhou H, Wu WKK, Wong SH, Coker OO, Dai Z, et al. Alterations in Enteric Virome Are Associated With Colorectal Cancer and Survival Outcomes. Gastroenterology. 2018;155(2):529-41.e5.
