# Supplementary material for: Expansion of Colorectal Cancer Biomarkers Based on Gut Bacteria and Viruses
Source: Cancers (Basel). 2022 Sep 25;14(19):4662. doi: 10.3390/cancers14194662 (PMC9563090; doi:10.3390/cancers14194662)
Supplement: Supplementary file 1 [file cancers-14-04662-s001.zip › Supplementary Tables S1¿CS7.pdf]

Supplementary Table S1. More clinical details on colorectal adenoma and colorectal cancer

Colorectal Adenoma		Colorectal Cancer	
**Location**	N(%)	**Location**	N (%)
colon	19 (65.52)	colon	16 (53.33)
rectum	8 (27.59)		
multiple locations	2 (6.90)	rectum	14 (46.67)
**Maximum diameter (d, cm)**			
d < 1	24 (82.76)	**AJCC Stage ***	
d ≥ 1	5 (17.24)	I	7 (23.33)
**Stage #**		II	10 (33.33)
non-advanced adenoma	19 (65.52)	III	11 (36.67)
advanced adenoma	10 (34.48)	IV	2 (6.67)

Note: # Stage: Advanced adenoma was defined as meeting any of the following conditions: villous or tubulovillous features, size ≥ 1 cm, or high-grade dysplasia. Non-adenomas were defined as those that did not meet all of the above criteria.

*AJCC Stage: The colorectal cancer stage was evaluated based on the American Joint Committee on Cancer (AJCC) TNM system. (https://www.cancer.org/cancer/colon-rectal-cancer/detection-diagnosis-staging/staged.html)

Supplementary Table S2. Bacterial species with statistical differences among the three groups

feature	Mean relative abundance in group			*P*-value	*P*adj-value
	Control	Adenoma	CRC		
Dialister pneumosintes	2.54×10^{-06}	0.00	4.82×10^{-04}	1.22×10^{-08}	3.10×10^{-06}
Clostridium symbiosum	1.28×10^{-05}	4.22×10^{-04}	2.50×10^{-03}	1.63×10^{-08}	3.10×10^{-06}
Clostridium bolteae	8.23×10^{-05}	5.38×10^{-03}	2.76×10^{-03}	2.06×10^{-08}	3.10×10^{-06}
Hungatella hathewayi	5.70×10^{-06}	1.46×10^{-03}	2.22×10^{-03}	2.17×10^{-08}	3.10×10^{-06}
Gemella morbillorum	7.76×10^{-06}	2.27×10^{-05}	7.15×10^{-04}	1.28×10^{-07}	1.28×10^{-05}
Peptostreptococcus stomatis	5.05×10^{-06}	3.17×10^{-05}	2.29×10^{-03}	1.34×10^{-07}	1.28×10^{-05}
Erysipelatoclostridium ramosum	2.74×10^{-05}	2.04×10^{-03}	1.06×10^{-03}	5.10×10^{-07}	3.95×10^{-05}
Slackia isoflavoniconvertens	9.62×10^{-04}	6.15×10^{-06}	7.03×10^{-07}	5.52×10^{-07}	3.95×10^{-05}
Parvimonas micra	1.68×10^{-06}	9.18×10^{-06}	1.34×10^{-03}	1.07×10^{-06}	6.80×10^{-05}
Bacteroides plebeius	2.26×10^{-02}	2.39×10^{-02}	9.42×10^{-03}	1.97×10^{-06}	1.13×10^{-04}
Ruthenibacterium lactatiformans	2.74×10^{-04}	4.19×10^{-03}	7.52×10^{-03}	3.76×10^{-06}	1.96×10^{-04}
Lactobacillus ruminis	2.20×10^{-02}	6.53×10^{-04}	1.23×10^{-03}	5.22×10^{-06}	2.49×10^{-04}
Megamonas funiformis	2.10×10^{-02}	8.67×10^{-04}	1.25×10^{-02}	7.62×10^{-06}	3.19×10^{-04}
Prevotella copri	1.98×10^{-01}	3.66×10^{-02}	6.43×10^{-02}	7.80×10^{-06}	3.19×10^{-04}
Bifidobacterium adolescentis	3.35×10^{-02}	1.78×10^{-02}	9.05×10^{-03}	9.81×10^{-06}	3.74×10^{-04}
Clostridium innocuum	4.55×10^{-04}	3.38×10^{-03}	2.23×10^{-03}	1.09×10^{-05}	3.90×10^{-04}
Fusobacterium nucleatum	0.00	1.67×10^{-03}	2.17×10^{-04}	1.55×10^{-05}	5.20×10^{-04}
Eggerthella lenta	8.65×10^{-04}	3.09×10^{-03}	4.74×10^{-03}	1.92×10^{-05}	6.09×10^{-04}
Coprococcus catus	1.96×10^{-03}	7.88×10^{-04}	8.61×10^{-04}	4.47×10^{-05}	1.35×10^{-03}
Bacteroides coprocola	1.41×10^{-02}	4.74×10^{-03}	2.36×10^{-03}	6.00×10^{-05}	1.68×10^{-03}
Bacteroides fragilis	1.48×10^{-02}	2.42×10^{-02}	2.95×10^{-02}	6.15×10^{-05}	1.68×10^{-03}
Bacteroides thetaiotaomicron	1.33×10^{-03}	3.54×10^{-02}	1.84×10^{-02}	6.50×10^{-05}	1.69×10^{-03}
Gemmiger formicilis	1.88×10^{-03}	9.62×10^{-04}	9.01×10^{-04}	7.47×10^{-05}	1.86×10^{-03}
Desulfovibrio piger	9.97×10^{-04}	5.64×10^{-05}	2.86×10^{-04}	8.14×10^{-05}	1.94×10^{-03}
Roseburia sp CAG 471	9.21×10^{-04}	2.66×10^{-04}	4.46×10^{-05}	1.17×10^{-04}	2.68×10^{-03}
Bilophila wadsworthia	7.78×10^{-05}	2.80×10^{-04}	5.20×10^{-04}	1.38×10^{-04}	3.03×10^{-03}
Ruminococcus gnavus	1.75×10^{-03}	1.66×10^{-02}	1.31×10^{-02}	1.45×10^{-04}	3.07×10^{-03}
Clostridium citroniae	5.88×10^{-06}	3.79×10^{-04}	1.44×10^{-04}	1.60×10^{-04}	3.27×10^{-03}
Barnesiella intestinihominis	6.37×10^{-03}	1.54×10^{-03}	5.93×10^{-04}	1.80×10^{-04}	3.49×10^{-03}
Firmicutes bacterium CAG 110	8.81×10^{-04}	2.69×10^{-05}	1.67×10^{-04}	1.88×10^{-04}	3.49×10^{-03}
Coprococcus eutactus	3.61×10^{-03}	2.08×10^{-03}	3.53×10^{-03}	1.89×10^{-04}	3.49×10^{-03}
Clostridium asparagiforme	8.32×10^{-08}	1.38×10^{-05}	1.51×10^{-04}	2.04×10^{-04}	3.64×10^{-03}
Clostridium clostridioforme	0.00	9.78×10^{-05}	7.09×10^{-04}	2.88×10^{-04}	4.99×10^{-03}
Clostridium aldenense	3.61×10^{-06}	3.43×10^{-05}	8.48×10^{-05}	3.01×10^{-04}	5.07×10^{-03}
Eikenella corrodens	0.00	1.06×10^{-04}	5.33×10^{-04}	4.40×10^{-04}	7.20×10^{-03}
Eubacterium rectale	5.26×10^{-02}	3.64×10^{-02}	2.03×10^{-02}	4.54×10^{-04}	7.22×10^{-03}
Clostridium scindens	0.00	6.59×10^{-05}	8.55×10^{-06}	4.80×10^{-04}	7.42×10^{-03}
Gordonibacter pamelaeae	1.76×10^{-04}	6.73×10^{-04}	8.48×10^{-04}	5.22×10^{-04}	7.85×10^{-03}
Blautia producta	0.00	8.60×10^{-05}	4.85×10^{-04}	7.37×10^{-04}	1.08×10^{-02}
Megamonas hypermegale	2.54×10^{-03}	2.06×10^{-04}	1.24×10^{-03}	9.01×10^{-04}	1.29×10^{-02}
Flavonifractor plautii	2.22×10^{-03}	6.38×10^{-03}	5.71×10^{-03}	9.65×10^{-04}	1.32×10^{-02}
Sellimonas intestinalis	0.00	3.28×10^{-04}	6.27×10^{-04}	9.67×10^{-04}	1.32×10^{-02}
Eubacterium siraeum	1.67×10^{-03}	2.93×10^{-03}	5.55×10^{-05}	1.08×10^{-03}	1.43×10^{-02}
Bacteroides coprophilus	9.79×10^{-04}	0.00	5.05×10^{-06}	1.14×10^{-03}	1.47×10^{-02}

Megasphaera elsdenii	2.30×10^{-03}	1.27×10^{-04}	6.10×10^{-05}	1.16×10^{-03}	1.47×10^{-02}
Oscillibacter sp CAG 241	5.99×10^{-04}	7.93×10^{-04}	7.20×10^{-04}	1.31×10^{-03}	1.62×10^{-02}
Paraprevotella xylaniphila	4.42×10^{-04}	7.76×10^{-04}	7.39×10^{-04}	1.33×10^{-03}	1.62×10^{-02}
Firmicutes bacterium CAG 170	1.36×10^{-03}	4.46×10^{-05}	1.94×10^{-03}	1.86×10^{-03}	2.21×10^{-02}
Blautia hansenii	1.79×10^{-05}	6.52×10^{-04}	1.52×10^{-04}	1.95×10^{-03}	2.25×10^{-02}
Phascolarctobacterium faecium	4.48×10^{-04}	3.74×10^{-03}	2.14×10^{-03}	1.97×10^{-03}	2.25×10^{-02}
Anaerotruncus colihominis	2.16×10^{-05}	1.90×10^{-04}	2.60×10^{-04}	2.02×10^{-03}	2.26×10^{-02}
Fusobacterium varium	1.83×10^{-06}	2.42×10^{-08}	8.64×10^{-04}	2.18×10^{-03}	2.40×10^{-02}
Roseburia intestinalis	9.38×10^{-03}	7.48×10^{-03}	7.17×10^{-04}	2.30×10^{-03}	2.49×10^{-02}
Faecalibacterium prausnitzii	4.64×10^{-02}	2.88×10^{-02}	2.09×10^{-02}	2.46×10^{-03}	2.60×10^{-02}
Lachnospira pectinoschiza	7.23×10^{-03}	1.69×10^{-03}	4.85×10^{-04}	2.66×10^{-03}	2.77×10^{-02}
Turicibacter sanguinis	2.93×10^{-04}	3.19×10^{-04}	3.34×10^{-05}	2.80×10^{-03}	2.86×10^{-02}
Oscillibacter sp 57 20	1.27×10^{-03}	7.89×10^{-04}	5.91×10^{-04}	3.00×10^{-03}	3.01×10^{-02}
Actinomyces sp HMSC035G02	1.75×10^{-05}	2.01×10^{-05}	4.14×10^{-05}	3.20×10^{-03}	3.16×10^{-02}
Tyzzerella nexilis	1.25×10^{-05}	9.22×10^{-04}	8.19×10^{-04}	3.61×10^{-03}	3.47×10^{-02}
Eisenbergiella massiliensis	0.00	4.30×10^{-04}	2.10×10^{-04}	3.71×10^{-03}	3.47×10^{-02}
Eubacterium sp CAG 38	1.23×10^{-03}	1.52×10^{-03}	8.76×10^{-04}	3.72×10^{-03}	3.47×10^{-02}
Fusobacterium sp oral taxon 370	0.00	0.00	8.43×10^{-05}	3.82×10^{-03}	3.47×10^{-02}
Prevotella intermedia	0.00	0.00	1.12×10^{-03}	3.82×10^{-03}	3.47×10^{-02}
Clostridium sp CAG 58	6.39×10^{-06}	1.70×10^{-04}	7.33×10^{-04}	3.92×10^{-03}	3.51×10^{-02}
Fusicatenibacter saccharivorans	2.08×10^{-02}	2.77×10^{-02}	9.62×10^{-03}	4.34×10^{-03}	3.82×10^{-02}
Holdemanella biformis	7.21×10^{-03}	8.05×10^{-05}	8.28×10^{-03}	4.83×10^{-03}	4.13×10^{-02}
Dialister succinatiphilus	4.32×10^{-03}	1.73×10^{-04}	4.81×10^{-04}	4.83×10^{-03}	4.13×10^{-02}
Blautia coccoides	0.00	3.84×10^{-05}	3.10×10^{-04}	5.34×10^{-03}	4.49×10^{-02}
Bacteroides uniformis	8.90×10^{-03}	2.68×10^{-02}	4.84×10^{-02}	5.84×10^{-03}	4.85×10^{-02}
Lactonifactor longoviformis	0.00	2.50×10^{-05}	2.02×10^{-05}	6.09×10^{-03}	4.98×10^{-02}
Eubacterium ramulus	9.95×10^{-04}	1.77×10^{-03}	7.80×10^{-04}	6.89×10^{-03}	5.49×10^{-02}
Catabacter hongkongensis	3.47×10^{-06}	3.42×10^{-05}	8.80×10^{-05}	6.92×10^{-03}	5.49×10^{-02}
Collinsella stercoris	7.67×10^{-05}	5.42×10^{-05}	3.50×10^{-05}	7.01×10^{-03}	5.49×10^{-02}
Coprobacillus cateniformis	2.95×10^{-07}	5.90×10^{-05}	1.80×10^{-04}	7.11×10^{-03}	5.50×10^{-02}
Ruminococcus bromii	2.08×10^{-02}	1.37×10^{-02}	2.11×10^{-02}	7.38×10^{-03}	5.63×10^{-02}
Parabacteroides distasonis	3.09×10^{-03}	1.51×10^{-02}	1.79×10^{-02}	8.31×10^{-03}	6.26×10^{-02}
Ruminococcus callidus	2.76×10^{-04}	1.26×10^{-04}	7.28×10^{-05}	9.02×10^{-03}	6.61×10^{-02}
Ruminococcus lactaris	1.59×10^{-03}	6.33×10^{-04}	7.61×10^{-04}	9.02×10^{-03}	6.61×10^{-02}
Roseburia faecis	2.97×10^{-02}	2.82×10^{-02}	9.04×10^{-03}	9.55×10^{-03}	6.92×10^{-02}
Ruminococcus sp CAG 579	8.84×10^{-04}	0.00	7.17×10^{-04}	9.92×10^{-03}	7.09×10^{-02}
Streptococcus mitis	3.07×10^{-05}	2.23×10^{-04}	3.94×10^{-04}	1.02×10^{-02}	7.19×10^{-02}
Coprococcus comes	3.78×10^{-03}	2.66×10^{-03}	2.82×10^{-03}	1.03×10^{-02}	7.19×10^{-02}
Streptococcus oralis	7.60×10^{-05}	3.11×10^{-04}	8.00×10^{-04}	1.05×10^{-02}	7.22×10^{-02}
Lactobacillus rogosae	1.60×10^{-04}	3.19×10^{-05}	2.65×10^{-06}	1.13×10^{-02}	7.71×10^{-02}
Clostridium bolteae CAG 59	2.33×10^{-05}	1.38×10^{-04}	1.41×10^{-04}	1.16×10^{-02}	7.83×10^{-02}
Enterococcus avium	0.00	3.27×10^{-04}	6.99×10^{-05}	1.20×10^{-02}	8.00×10^{-02}
Campylobacter showae	0.00	0.00	1.31×10^{-05}	1.22×10^{-02}	8.02×10^{-02}
Eubacterium sp CAG 251	5.55×10^{-03}	3.22×10^{-03}	1.36×10^{-03}	1.30×10^{-02}	8.47×10^{-02}
Bacteroides dorei	1.45×10^{-03}	1.01×10^{-02}	2.28×10^{-02}	1.32×10^{-02}	8.51×10^{-02}
Fusobacterium mortiferum	6.90×10^{-05}	2.28×10^{-03}	9.00×10^{-03}	1.57×10^{-02}	1.00×10^{-01}
Anaerococcus vaginalis	0.00	6.08×10^{-07}	3.25×10^{-05}	1.69×10^{-02}	1.05×10^{-01}
Enorma massiliensis	0.00	2.09×10^{-06}	7.56×10^{-05}	1.69×10^{-02}	1.05×10^{-01}

Bacteroides finegoldii	9.39×10^{-04}	5.69×10^{-04}	1.56×10^{-03}	1.73×10^{-02}	1.06×10^{-01}
Mitsuokella multacida	2.39×10^{-03}	2.54×10^{-05}	0.00	1.82×10^{-02}	1.09×10^{-01}
Ruminococcus sp CAG 488	1.10×10^{-03}	0.00	3.92×10^{-05}	1.83×10^{-02}	1.09×10^{-01}
Blautia sp CAG 257	1.48×10^{-05}	1.80×10^{-03}	3.81×10^{-04}	1.84×10^{-02}	1.09×10^{-01}
Eisenbergiella tayi	3.29×10^{-05}	1.30×10^{-04}	6.39×10^{-05}	2.01×10^{-02}	1.18×10^{-01}
Collinsella aerofaciens	2.36×10^{-02}	2.22×10^{-02}	1.54×10^{-02}	2.12×10^{-02}	1.24×10^{-01}
Prevotella sp CAG 279	1.16×10^{-02}	3.27×10^{-03}	4.00×10^{-03}	2.23×10^{-02}	1.29×10^{-01}
Eubacterium ventriosum	1.45×10^{-03}	2.74×10^{-03}	8.25×10^{-04}	2.28×10^{-02}	1.31×10^{-01}
Phascolarctobacterium succinatutens	4.61×10^{-03}	1.27×10^{-04}	3.03×10^{-04}	2.32×10^{-02}	1.31×10^{-01}
Dorea longicatena	1.55×10^{-02}	8.27×10^{-03}	7.32×10^{-03}	2.61×10^{-02}	1.46×10^{-01}
Streptococcus sanguinis	7.57×10^{-05}	1.31×10^{-04}	8.84×10^{-05}	2.64×10^{-02}	1.47×10^{-01}
Streptococcus cristatus	2.32×10^{-06}	3.03×10^{-05}	1.90×10^{-04}	2.69×10^{-02}	1.48×10^{-01}
Bacteroides xylanisolvens	8.61×10^{-04}	5.39×10^{-03}	7.94×10^{-03}	2.71×10^{-02}	1.48×10^{-01}
Butyricimonas synergistica	1.96×10^{-04}	4.24×10^{-05}	1.46×10^{-04}	2.81×10^{-02}	1.51×10^{-01}
Clostridium sp CAG 242	0.00	1.31×10^{-04}	3.49×10^{-04}	2.83×10^{-02}	1.51×10^{-01}
Roseburia inulinivorans	1.22×10^{-02}	1.06×10^{-02}	9.61×10^{-03}	2.97×10^{-02}	1.57×10^{-01}
Leclercia adecarboxylata	1.24×10^{-05}	0.00	0.00	3.08×10^{-02}	1.61×10^{-01}
Clostridium butyricum	0.00	1.31×10^{-04}	0.00	3.22×10^{-02}	1.66×10^{-01}
Enterococcus asini	0.00	4.66×10^{-04}	0.00	3.22×10^{-02}	1.66×10^{-01}
Enterococcus faecalis	4.70×10^{-05}	2.46×10^{-04}	3.61×10^{-05}	3.33×10^{-02}	1.70×10^{-01}
Actinomyces sp S6 Spd3	2.19×10^{-06}	8.01×10^{-06}	4.84×10^{-06}	3.36×10^{-02}	1.70×10^{-01}
Granulicatella adiacens	0.00	5.67×10^{-07}	2.26×10^{-05}	3.41×10^{-02}	1.71×10^{-01}
Parabacteroides merdae	4.94×10^{-03}	3.36×10^{-03}	3.99×10^{-03}	3.52×10^{-02}	1.75×10^{-01}
Eubacterium eligens	1.54×10^{-03}	3.16×10^{-03}	4.81×10^{-03}	3.57×10^{-02}	1.76×10^{-01}
Blautia obeum	5.16×10^{-03}	3.27×10^{-03}	6.49×10^{-03}	3.68×10^{-02}	1.80×10^{-01}
Roseburia sp CAG 182	5.63×10^{-04}	4.20×10^{-05}	1.62×10^{-04}	3.76×10^{-02}	1.82×10^{-01}
Aeromonas veronii	0.00	0.00	1.51×10^{-05}	3.80×10^{-02}	1.83×10^{-01}
Coprobacter fastidiosus	7.34×10^{-04}	1.34×10^{-04}	3.38×10^{-05}	3.86×10^{-02}	1.83×10^{-01}
Ruminococcus torques	6.58×10^{-03}	1.57×10^{-02}	4.88×10^{-03}	3.87×10^{-02}	1.83×10^{-01}
Odoribacter splanchnicus	3.25×10^{-03}	1.49×10^{-03}	4.40×10^{-03}	3.90×10^{-02}	1.83×10^{-01}
Olsenella scatoligenes	2.53×10^{-04}	1.59×10^{-04}	7.57×10^{-05}	3.99×10^{-02}	1.84×10^{-01}
Eubacterium limosum	3.12×10^{-06}	1.00×10^{-05}	4.39×10^{-05}	4.00×10^{-02}	1.84×10^{-01}
Eubacterium sp CAG 274	4.84×10^{-03}	1.98×10^{-03}	7.67×10^{-04}	4.01×10^{-02}	1.84×10^{-01}
Allisonella histaminiformans	1.64×10^{-04}	3.02×10^{-05}	4.96×10^{-06}	4.11×10^{-02}	1.87×10^{-01}
Bacteroides salyersiae	2.67×10^{-04}	3.16×10^{-04}	2.60×10^{-03}	4.25×10^{-02}	1.91×10^{-01}
Bifidobacterium breve	8.51×10^{-07}	3.87×10^{-05}	1.69×10^{-03}	4.32×10^{-02}	1.93×10^{-01}
Streptococcus thermophilus	2.15×10^{-06}	1.03×10^{-03}	1.96×10^{-04}	4.36×10^{-02}	1.93×10^{-01}
Clostridium leptum	1.04×10^{-05}	8.80×10^{-05}	3.15×10^{-04}	4.39×10^{-02}	1.93×10^{-01}
Parabacteroides johnsonii	1.61×10^{-04}	0.00	6.71×10^{-04}	4.42×10^{-02}	1.93×10^{-01}
Prevotella stercorea	1.42×10^{-02}	3.56×10^{-03}	6.48×10^{-03}	4.52×10^{-02}	1.96×10^{-01}
Catenibacterium mitsuokai	1.06×10^{-03}	6.25×10^{-05}	3.73×10^{-03}	4.59×10^{-02}	1.97×10^{-01}
Ruminococcus sp CAG 403	1.26×10^{-03}	0.00	6.47×10^{-04}	4.61×10^{-02}	1.97×10^{-01}
Oxalobacter formigenes	0.00	1.27×10^{-04}	6.68×10^{-05}	4.69×10^{-02}	1.99×10^{-01}
Proteobacteria bacterium CAG 139	3.08×10^{-04}	2.04×10^{-03}	4.89×10^{-03}	4.79×10^{-02}	2.02×10^{-01}
Peptoniphilus harei	0.00	5.56×10^{-07}	3.79×10^{-05}	4.96×10^{-02}	2.06×10^{-01}
Absiella dolichum	0.00	7.82×10^{-05}	1.18×10^{-04}	4.97×10^{-02}	2.06×10^{-01}

Note: Using the Kruskal-Wallis test to identify the differentially expressed bacterial species among control, adenoma, and CRC groups. *P*-adj was obtained by BH correction of *P* value.

Supplementary Table S3. Bacterial species with statistical differences between two comparisons

feature	Adenoma vs Control	CRC vs Control	CRC vs Adenoma	feature	Adenoma vs Control	CRC vs Control	CRC vs Adenoma
Dialister pneumosintes	/	↑	↑	Lactonifactor longoviformis	↑	↑	/
Clostridium symbiosum	↑	↑	↑	Eubacterium ramulus	↓	↓	/
Clostridium bolteae	↑	↑	/	Catabacter hongkongensis	↑	↑	/
Hungatella hathewayi	↑	↑	/	Collinsella stercoris	↓	↓	/
Gemella morbillorum	/	↑	↑	Coprobacillus cateniformis	↑	↑	/
Peptostreptococcus stomatis	/	↑	↑	Ruminococcus bromii	↓	/	/
Erysipelatoclostridium ramosum	↑	↑	/	Parabacteroides distasonis	↑	↑	/
Slackia isoflavoniconvertens	↓	↓	/	Ruminococcus callidus	↓	↓	/
Parvimonas micra	/	↑	↑	Ruminococcus lactaris	↓	↓	/
Bacteroides plebeius	↓	↓	/	Roseburia faecis	↓	↓	/
Ruthenibacterium lactatiformans	↑	↑	/	Ruminococcus sp CAG 579	↓	/	/
Lactobacillus ruminis	↓	↓	/	Streptococcus mitis	↑	↑	/
Megamonas funiformis	↓	↓	/	Coprococcus comes	↓	↓	/
Prevotella copri	↓	↓	/	Streptococcus oralis	/	↑	↑
Bifidobacterium adolescentis	↓	↓	/	Lactobacillus rogosae	/	↓	/
Clostridium innocuum	↑	↑	/	Clostridium bolteae CAG 59	↑	↑	/
Fusobacterium nucleatum	/	↑	↑	Enterococcus avium	↑	↑	/
Eggerthella lenta	↑	↑	/	Campylobacter showae	NA	↑	↑
Coprococcus catus	↓	↓	/	Eubacterium sp CAG 251	↓	↓	/
Bacteroides coprocola	↓	↓	/	Bacteroides dorei	↑	↑	/
Bacteroides fragilis	↑	↑	↑	Fusobacterium mortiferum	/	↑	/
Bacteroides thetaiotaomicron	↑	↑	/	Anaerococcus vaginalis	/	↑	/
Gemmiger formicilis	↓	↓	/	Enorma massiliensis	/	↑	/
Desulfovibrio piger	↓	↓	/	Bacteroides finegoldii	↓	/	/
Roseburia sp CAG 471	↓	↓	/	Mitsuokella multacida	/	↓	/
Bilophila wadsworthia	↑	↑	↑	Ruminococcus sp CAG 488	↓	/	/
Ruminococcus gnavus	↑	↑	/	Blautia sp CAG 257	↑	↑	/

Clostridium citroniae	↑	↑	/	Eisenbergiella tayi	↑	↑	/
Barnesiella intestinihominis	↓	↓	/	Collinsella aerofaciens	/	↓	/
Firmicutes bacterium CAG 110	↓	↓	/	Prevotella sp CAG 279	↓	/	/
Coprococcus eutactus	↓	↓	/	Eubacterium ventriosum	/	↓	/
Clostridium asparagiforme	/	↑	↑	Phascolarctobacterium succinatutens	↓	/	/
Clostridium clostridioforme	↑	↑	/	Dorea longicatena	↓	↓	/
Clostridium aldenense	↑	↑	/	Streptococcus sanguinis	↑	/	/
Eikenella corrodens	/	↑	↑	Streptococcus cristatus	/	↑	/
Eubacterium rectale	↓	↓	/	Bacteroides xylanisolvens	/	↑	/
Clostridium scindens	↑	↑	/	Butyricimonas synergistica	↓	/	/
Gordonibacter pamelaeae	↑	↑	/	Clostridium sp CAG 242	↑	↑	/
Blautia producta	↑	↑	/	Roseburia inulinivorans	/	↓	/
Megamonas hypermegale	↓	↓	/	Leclercia adecarboxylata	/	/	NA
Flavonifractor plautii	↑	↑	/	Clostridium butyricum	/	NA	/
Sellimonas intestinalis	↑	↑	/	Enterococcus asini	/	NA	/
Eubacterium siraeum	↓	↓	/	Enterococcus faecalis	↑	/	/
Bacteroides coprophilus	↓	↓	/	Actinomyces sp S6 Spd3	↑	↑	/
Megasphaera elsdenii	↓	↓	/	Granulicatella adiacens	/	↑	/
Oscillibacter sp CAG 241	↓	↓	/	Parabacteroides merdae	↓	/	/
Paraprevotella xylaniphila	↓	↓	/	Eubacterium eligens	↓	↓	/
Firmicutes bacterium CAG 170	↓	/	/	Blautia obeum	↓	↓	/
Blautia hansenii	↑	/	↓	Roseburia sp CAG 182	↓	/	/
Phascolarctobacterium faecium	↑	↑	/	Aeromonas veronii	NA	/	/
Anaerotruncus colihominis	↑	↑	/	Coprobacter fastidiosus	/	/	/
Fusobacterium varium	/	↑	↑	Ruminococcus torques	/	↓	/
Roseburia intestinalis	/	↓	/	Odoribacter splanchnicus	↓	/	↑
Faecalibacterium prausnitzii	↓	↓	/	Olsenella scatoligenes	↓	/	/
Lachnospira pectinoschiza	↓	↓	/	Eubacterium limosum	/	↑	/
Turicibacter sanguinis	/	↓	↓	Eubacterium sp CAG 274	↓	↓	/
Oscillibacter sp 57 20	↓	/	/	Allisonella histaminiformans	↓	↓	/
Actinomyces sp HMSC035G02	↑	↑	/	Bacteroides salyersiae	/	↑	/
Tyzzerella nexilis	↑	↑	/	Bifidobacterium breve	/	↑	/

Eisenbergiella massiliensis	↑	↑	/	Streptococcus thermophilus	/	↑	/
Eubacterium sp CAG 38	↓	↓	/	Clostridium leptum	↑	↑	/
Fusobacterium sp oral taxon 370	NA	↑	↑	Parabacteroides johnsonii	↓	/	↑
Prevotella intermedia	NA	↑	↑	Prevotella stercorea	↓	/	/
Clostridium sp CAG 58	/	↑	↑	Catenibacterium mitsuokai	↓	/	/
Fusicatenibacter saccharivorans	/	↓	/	Ruminococcus sp CAG 4/3	↓	/	/
Holdemanella biformis	↓	/	/	Oxalobacter formigenes	↑	/	/
Dialister succinatiphilus	↓	↓	/	Proteobacteria bacterium CAG 139	↑	↑	/
Blautia coccoides	↑	↑	/	Peptoniphilus harei	/	↑	/
Bacteroides uniformis	↑	↑	/	Absiella dolichum	↑	/	/

Note: Wilcoxon rank sum test and BH correction were used for comparison between every two groups. Bacterial species that are statistically different between the two groups compared with *P*-adj values < 0.05. ↑indicates a significant increase in the pre-vs group compared to the post-vs group. ↓indicates a significant decrease in the pre-vs group compared to the post-vs group. / indicates no statistical difference. NA indicates that the species was not annotated in all samples of a given group in the two groups being compared.

Supplementary Table S4. KEGG metabolic pathways that are statistically different between the three groups (control, adenoma, and CRC)

feature	annotation	Mean relative abundance in group			*P*-value	*P*adj-value
		Control	Adenoma	CRC		
ko00051	Fructose and mannose metabolism	5.04×10^{-03}	6.03×10^{-03}	5.70×10^{-03}	6.99×10^{-04}	9.66×10^{-03}
ko00052	Galactose metabolism	3.20×10^{-03}	3.91×10^{-03}	3.94×10^{-03}	4.41×10^{-04}	8.35×10^{-03}
ko00053	Ascorbate and aldarate metabolism	8.06×10^{-04}	1.26×10^{-03}	8.45×10^{-04}	5.25×10^{-03}	3.43×10^{-02}
ko00140	Steroid hormone biosynthesis	2.05×10^{-04}	3.60×10^{-04}	4.66×10^{-04}	4.60×10^{-04}	8.35×10^{-03}
ko00190	Oxidative phosphorylation	1.18×10^{-02}	1.43×10^{-02}	1.49×10^{-02}	1.17×10^{-04}	4.85×10^{-03}
ko00195	Photosynthesis	1.45×10^{-02}	1.32×10^{-02}	1.24×10^{-02}	2.48×10^{-03}	2.35×10^{-02}
ko00220	Arginine biosynthesis	6.19×10^{-03}	5.42×10^{-03}	5.48×10^{-03}	9.54×10^{-05}	4.53×10^{-03}
ko00230	Purine metabolism	2.37×10^{-02}	2.28×10^{-02}	2.31×10^{-02}	2.02×10^{-03}	1.98×10^{-02}
ko00240	Pyrimidine metabolism	1.97×10^{-02}	1.79×10^{-02}	1.87×10^{-02}	3.19×10^{-04}	8.35×10^{-03}
ko00253	Tetracycline biosynthesis	3.57×10^{-05}	5.58×10^{-06}	2.13×10^{-05}	5.77×10^{-04}	8.35×10^{-03}
ko00310	Lysine degradation	1.04×10^{-03}	1.29×10^{-03}	1.34×10^{-03}	8.89×10^{-03}	4.68×10^{-02}
ko00330	Arginine and proline metabolism	6.96×10^{-03}	6.14×10^{-03}	6.73×10^{-03}	1.19×10^{-03}	1.52×10^{-02}
ko00361	Chlorocyclohexane and chlorobenzene degradation	9.80×10^{-06}	3.76×10^{-07}	3.12×10^{-08}	6.11×10^{-03}	3.76×10^{-02}
ko00380	Tryptophan metabolism	1.69×10^{-04}	2.82×10^{-04}	2.87×10^{-04}	2.60×10^{-04}	8.34×10^{-03}
ko00400	Phenylalanine, tyrosine and tryptophan biosynthesis	1.40×10^{-02}	1.28×10^{-02}	1.26×10^{-02}	4.54×10^{-03}	3.26×10^{-02}
ko00460	Cyanoamino acid metabolism	1.77×10^{-03}	2.04×10^{-03}	2.07×10^{-03}	5.68×10^{-03}	3.62×10^{-02}
ko00472	D-Arginine and D-ornithine metabolism	1.17×10^{-05}	1.62×10^{-05}	1.66×10^{-05}	7.27×10^{-03}	4.07×10^{-02}
ko00511	Other glycan degradation	2.85×10^{-03}	4.05×10^{-03}	4.28×10^{-03}	1.42×10^{-03}	1.60×10^{-02}
ko00524	Neomycin, kanamycin and gentamicin biosynthesis	2.66×10^{-03}	3.09×10^{-03}	3.03×10^{-03}	6.49×10^{-05}	3.59×10^{-03}
ko00563	Glycosylphosphatidylinositol (GPI)-anchor biosynthesis	2.75×10^{-05}	9.16×10^{-05}	7.42×10^{-05}	5.86×10^{-03}	3.67×10^{-02}
ko00564	Glycerophospholipid metabolism	1.37×10^{-02}	1.34×10^{-02}	1.24×10^{-02}	3.85×10^{-04}	8.35×10^{-03}
ko00620	Pyruvate metabolism	7.03×10^{-03}	8.01×10^{-03}	8.29×10^{-03}	4.31×10^{-03}	3.18×10^{-02}
ko00625	Chloroalkane and chloroalkene degradation	6.91×10^{-04}	9.00×10^{-04}	8.87×10^{-04}	3.13×10^{-03}	2.51×10^{-02}
ko00633	Nitrotoluene degradation	4.64×10^{-04}	6.64×10^{-04}	6.50×10^{-04}	8.61×10^{-03}	4.68×10^{-02}
ko00640	Propanoate metabolism	4.54×10^{-03}	5.52×10^{-03}	5.60×10^{-03}	1.32×10^{-04}	4.87×10^{-03}
ko00900	Terpenoid backbone biosynthesis	1.10×10^{-02}	9.99×10^{-03}	1.01×10^{-02}	5.50×10^{-04}	8.35×10^{-03}
ko00966	Glucosinolate biosynthesis	2.02×10^{-03}	1.96×10^{-03}	1.87×10^{-03}	2.88×10^{-03}	2.51×10^{-02}
ko00980	Metabolism of xenobiotics by cytochrome P450	2.52×10^{-06}	5.07×10^{-06}	6.09×10^{-06}	4.77×10^{-03}	3.30×10^{-02}

ko00999	Biosynthesis of various secondary metabolites - part 1	9.03×10^{-06}	3.76×10^{-06}	3.15×10^{-06}	1.36×10^{-03}	1.60×10^{-02}
ko01057	Biosynthesis of type II polyketide products	4.99×10^{-06}	8.48×10^{-06}	9.77×10^{-06}	2.76×10^{-04}	8.34×10^{-03}
ko02030	Bacterial chemotaxis	5.58×10^{-03}	5.52×10^{-03}	3.85×10^{-03}	7.19×10^{-03}	4.07×10^{-02}
ko02040	Flagellar assembly	6.25×10^{-03}	6.14×10^{-03}	3.63×10^{-03}	4.16×10^{-03}	3.14×10^{-02}
ko03030	DNA replication	4.31×10^{-03}	3.78×10^{-03}	3.89×10^{-03}	8.90×10^{-04}	1.18×10^{-02}
ko03320	PPAR signaling pathway	3.26×10^{-04}	3.99×10^{-04}	5.02×10^{-04}	7.05×10^{-03}	4.07×10^{-02}
ko03420	Nucleotide excision repair	4.58×10^{-03}	4.19×10^{-03}	4.08×10^{-03}	5.58×10^{-04}	8.35×10^{-03}
ko03440	Homologous recombination	2.72×10^{-02}	2.55×10^{-02}	2.60×10^{-02}	2.84×10^{-03}	2.51×10^{-02}
ko03450	Non-homologous end-joining	4.39×10^{-05}	1.52×10^{-04}	1.81×10^{-04}	5.04×10^{-05}	3.35×10^{-03}
ko04010	MAPK signaling pathway	6.32×10^{-06}	0.00	7.77×10^{-08}	4.58×10^{-04}	8.35×10^{-03}
ko04070	Phosphatidylinositol signaling system	1.22×10^{-03}	1.29×10^{-03}	1.39×10^{-03}	2.85×10^{-03}	2.51×10^{-02}
ko04114	Oocyte meiosis	1.26×10^{-06}	0.00	1.25×10^{-06}	1.48×10^{-03}	1.60×10^{-02}
ko04122	Sulfur relay system	1.18×10^{-02}	1.17×10^{-02}	1.02×10^{-02}	9.03×10^{-03}	4.68×10^{-02}
ko04142	Lysosome	2.46×10^{-03}	3.39×10^{-03}	4.18×10^{-03}	3.17×10^{-03}	2.51×10^{-02}
ko04270	Vascular smooth muscle contraction	2.24×10^{-06}	5.14×10^{-07}	1.00×10^{-06}	3.81×10^{-04}	8.35×10^{-03}
ko04361	Axon regeneration	9.45×10^{-06}	1.12×10^{-06}	9.96×10^{-06}	5.26×10^{-03}	3.43×10^{-02}
ko04640	Hematopoietic cell lineage	5.27×10^{-06}	1.64×10^{-06}	1.05×10^{-06}	1.15×10^{-05}	1.70×10^{-03}
ko04722	Neurotrophin signaling pathway	1.36×10^{-07}	5.86×10^{-06}	4.07×10^{-06}	2.04×10^{-06}	6.78×10^{-04}
ko04915	Estrogen signaling pathway	2.07×10^{-07}	8.56×10^{-07}	1.50×10^{-06}	5.03×10^{-05}	3.35×10^{-03}
ko04919	Thyroid hormone signaling pathway	7.57×10^{-05}	1.68×10^{-04}	1.01×10^{-04}	4.62×10^{-03}	3.26×10^{-02}
ko04921	Oxytocin signaling pathway	2.08×10^{-06}	1.30×10^{-06}	3.18×10^{-07}	3.25×10^{-03}	2.51×10^{-02}
ko04924	Renin secretion	1.75×10^{-06}	6.09×10^{-07}	1.22×10^{-06}	7.25×10^{-03}	4.07×10^{-02}
ko04940	Type I diabetes mellitus	1.93×10^{-04}	3.10×10^{-04}	3.43×10^{-04}	7.36×10^{-03}	4.07×10^{-02}
ko04950	Maturity onset diabetes of the young	4.46×10^{-06}	1.23×10^{-06}	4.89×10^{-06}	5.49×10^{-04}	8.35×10^{-03}
ko04971	Gastric acid secretion	4.14×10^{-05}	2.25×10^{-05}	2.75×10^{-05}	1.54×10^{-03}	1.60×10^{-02}
ko05016	Huntington disease	4.53×10^{-04}	7.19×10^{-04}	7.33×10^{-04}	1.93×10^{-03}	1.94×10^{-02}
ko05134	Legionellosis	2.28×10^{-03}	2.10×10^{-03}	1.92×10^{-03}	5.79×10^{-04}	8.35×10^{-03}
ko05152	Tuberculosis	3.57×10^{-03}	3.46×10^{-03}	3.28×10^{-03}	3.03×10^{-03}	2.51×10^{-02}
ko05165	Human papillomavirus infection	1.01×10^{-05}	4.08×10^{-06}	5.11×10^{-06}	6.27×10^{-03}	3.78×10^{-02}
ko05202	Transcriptional misregulation in cancer	9.92×10^{-05}	2.97×10^{-05}	4.35×10^{-05}	5.52×10^{-04}	8.35×10^{-03}
ko05219	Bladder cancer	1.60×10^{-03}	1.33×10^{-03}	1.37×10^{-03}	5.24×10^{-03}	3.43×10^{-02}
ko05225	Hepatocellular carcinoma	1.27×10^{-05}	6.05×10^{-06}	6.95×10^{-06}	1.54×10^{-03}	1.60×10^{-02}

ko05226	Gastric cancer	5.92×10^{-06}	4.17×10^{-06}	2.92×10^{-06}	1.29×10^{-03}	1.59×10^{-02}
ko05230	Central carbon metabolism in cancer	6.04×10^{-03}	6.50×10^{-03}	6.86×10^{-03}	3.17×10^{-03}	2.51×10^{-02}
ko05235	PD-L1 expression and PD-1 checkpoint pathway in cancer	6.55×10^{-06}	2.99×10^{-05}	2.81×10^{-05}	1.53×10^{-05}	1.70×10^{-03}
ko05414	Dilated cardiomyopathy (DCM)	4.31×10^{-06}	7.95×10^{-07}	1.66×10^{-06}	8.90×10^{-03}	4.68×10^{-02}

Note: Using the Kruskal-Wallis test to identify the differentially expressed pathways among control, adenoma, and CRC groups. *P*-adj was obtained by BH correction of *P* value.

Supplementary Table S5. KEGG metabolic pathways with statistical differences between two comparisons

feature	annotation	Adenoma vs Control	CRC vs Control	Adenoma vs CRC
ko00051	Fructose and mannose metabolism	↑	↑	/
ko00052	Galactose metabolism	↑	↑	/
ko00053	Ascorbate and aldarate metabolism	↑	↑	/
ko00140	Steroid hormone biosynthesis	↑	↑	/
ko00190	Oxidative phosphorylation	↑	↑	/
ko00195	Photosynthesis	/	↓	/
ko00220	Arginine biosynthesis	↓	↓	/
ko00230	Purine metabolism	↓	↓	/
ko00240	Pyrimidine metabolism	↓	↓	/
ko00253	Tetracycline biosynthesis	↓	↓	/
ko00310	Lysine degradation	↑	↑	/
ko00330	Arginine and proline metabolism	↓	/	/
ko00361	Chlorocyclohexane and chlorobenzene degradation	↓	↓	/
ko00380	Tryptophan metabolism	↓	↓	/
ko00400	Phenylalanine, tyrosine and tryptophan biosynthesis	↓	↓	/
ko00460	Cyanoamino acid metabolism	/	↑	/
ko00472	D-Arginine and D-ornithine metabolism	↑	↑	/
ko00511	Other glycan degradation	↑	↑	/
ko00524	Neomycin, kanamycin and gentamicin biosynthesis	↑	↑	/
ko00563	Glycosylphosphatidylinositol (GPI)-anchor biosynthesis	↓	↓	/
ko00564	Glycerophospholipid metabolism	/	↓	/
ko00620	Pyruvate metabolism	↑	↑	/
ko00625	Chloroalkane and chloroalkene degradation	↑	↑	/
ko00633	Nitrotoluene degradation	↑	↑	/
ko00640	Propanoate metabolism	↑	↑	/
ko00900	Terpenoid backbone biosynthesis	↓	↓	/
ko00966	Glucosinolate biosynthesis	/	↓	/
ko00980	Metabolism of xenobiotics by cytochrome P450	/	↑	/
ko00999	Biosynthesis of various secondary metabolites - part 1	↓	↓	/

ko01057	Biosynthesis of type II polyketide products	↑	↑	/
ko02030	Bacterial chemotaxis	/	↓	/
ko02040	Flagellar assembly	/	↓	/
ko03030	DNA replication	↓	↓	/
ko03320	PPAR signaling pathway	/	↑	/
ko03420	Nucleotide excision repair	↓	↓	/
ko03440	Homologous recombination	↓	/	/
ko03450	Non-homologous end-joining	↓	↓	/
ko04010	MAPK signaling pathway	↓	↓	/
ko04070	Phosphatidylinositol signaling system	/	↑	/
ko04114	Oocyte meiosis	↓	/	/
ko04122	Sulfur relay system	/	↓	/
ko04142	Lysosome	/	↑	/
ko04270	Vascular smooth muscle contraction	↓	↓	/
ko04361	Axon regeneration	↓	/	/
ko04640	Hematopoietic cell lineage	↓	↓	/
ko04722	Neurotrophin signaling pathway	↑	↑	/
ko04915	Estrogen signaling pathway	↑	↑	/
ko04919	Thyroid hormone signaling pathway	↓	↓	/
ko04921	Oxytocin signaling pathway	↓	↓	/
ko04924	Renin secretion	↓	↓	/
ko04940	Type I diabetes mellitus	↑	↑	/
ko04950	Maturity onset diabetes of the young	↓	/	/
ko04971	Gastric acid secretion	↓	↑	/
ko05016	Huntington disease	↑	↑	/
ko05134	Legionellosis	/	↓	/
ko05152	Tuberculosis	/	↓	/
ko05165	Human papillomavirus infection	↓	↓	/
ko05202	Transcriptional misregulation in cancer	↓	↓	/
ko05219	Bladder cancer	↓	↓	/
ko05225	Hepatocellular carcinoma	↓	↓	/
ko05226	Gastric cancer	↓	↓	/

ko05230	Central carbon metabolism in cancer	/	↑	/
ko05235	PD-L1 expression and PD-1 checkpoint pathway in cancer	↑	↑	/
ko05414	Dilated cardiomyopathy (DCM)	↓	↓	/

Note: Wilcoxon rank sum test and BH correction were used for comparison between every two groups. Pathways that are statistically different between the two groups compared with *P*-adj values < 0.05. ↑indicates a significant increase in the pre-vs group compared to the post-vs group. ↓indicates a significant decrease in the pre-vs group compared to the post-vs group. / indicates no statistical difference.

Supplementary Table S6. Viral species with statistical differences among the three groups

feature	Mean relative abundance in group			*P*-value	*P*adj-value
	Control	Adenoma	CRC		
Phage FAKO27_000271F	2.64×10^{-02}	1.40×10^{-03}	1.79×10^{-03}	5.73×10^{-07}	1.07×10^{-03}
Faecalibacterium virus Lugh	4.50×10^{-03}	7.26×10^{-04}	1.21×10^{-03}	2.98×10^{-06}	2.79×10^{-03}
Faecalibacterium virus Toutatis	5.14×10^{-03}	9.92×10^{-04}	9.04×10^{-04}	6.20×10^{-06}	3.88×10^{-03}
Faecalibacterium virus Oengus	6.09×10^{-03}	6.06×10^{-03}	1.96×10^{-03}	4.40×10^{-05}	2.06×10^{-02}
uncultured phage	4.18×10^{-03}	2.50×10^{-03}	1.86×10^{-03}	3.15×10^{-04}	7.38×10^{-02}
uncultured Caudovirales phage	5.65×10^{-03}	2.03×10^{-03}	3.12×10^{-03}	7.58×10^{-04}	1.29×10^{-01}
Microvirus sp.	8.87×10^{-05}	0.00	6.15×10^{-06}	1.14×10^{-03}	1.78×10^{-01}
Bacillus virus G	6.15×10^{-05}	5.36×10^{-06}	6.37×10^{-06}	1.39×10^{-03}	2.01×10^{-01}
Faecalibacterium virus Brigit	7.06×10^{-03}	1.84×10^{-02}	1.05×10^{-03}	2.52×10^{-03}	2.95×10^{-01}
Streptococcus phage CHPC1042	0.00	0.00	5.81×10^{-05}	3.82×10^{-03}	3.41×10^{-01}
Streptococcus satellite phage Javan434	0.00	0.00	1.38×10^{-04}	3.82×10^{-03}	3.41×10^{-01}
unidentified virus	0.00	0.00	2.94×10^{-03}	3.82×10^{-03}	3.41×10^{-01}
CrAssphage sp.	3.22×10^{-03}	2.63×10^{-04}	4.18×10^{-05}	4.07×10^{-03}	3.42×10^{-01}
Faecalibacterium virus Epona	4.60×10^{-03}	1.42×10^{-04}	7.58×10^{-03}	4.20×10^{-03}	3.42×10^{-01}
Acidovorax virus ACP17	9.55×10^{-05}	0.00	0.00	4.81×10^{-03}	3.61×10^{-01}
Streptomyces phage Kardashian	1.41×10^{-04}	0.00	0.00	4.81×10^{-03}	3.61×10^{-01}
Streptococcus phage Javan326	0.00	6.88×10^{-06}	3.68×10^{-04}	6.04×10^{-03}	4.20×10^{-01}
Streptococcus phage Javan316	1.49×10^{-06}	1.31×10^{-05}	2.06×10^{-04}	6.04×10^{-03}	4.20×10^{-01}
Streptococcus phage Javan191	2.05×10^{-04}	9.83×10^{-05}	6.03×10^{-05}	7.22×10^{-03}	4.52×10^{-01}
Streptococcus phage Javan110	3.70×10^{-05}	1.76×10^{-05}	6.36×10^{-04}	7.23×10^{-03}	4.52×10^{-01}
Streptococcus phage Javan59	2.45×10^{-05}	3.70×10^{-05}	9.67×10^{-04}	8.82×10^{-03}	4.90×10^{-01}
Erwinia virus Wellington	3.38×10^{-06}	3.34×10^{-05}	3.62×10^{-05}	8.84×10^{-03}	4.90×10^{-01}
Streptococcus satellite phage Javan540	1.50×10^{-05}	1.47×10^{-04}	5.00×10^{-04}	9.69×10^{-03}	4.90×10^{-01}
Microviridae sp.	2.25×10^{-04}	1.86×10^{-04}	1.03×10^{-04}	1.15×10^{-02}	4.90×10^{-01}
Streptococcus phage Javan318	0.00	3.22×10^{-04}	2.16×10^{-04}	1.16×10^{-02}	4.90×10^{-01}
Streptococcus phage IPP25	0.00	0.00	6.12×10^{-05}	1.22×10^{-02}	4.90×10^{-01}
Streptococcus phage IPP27	0.00	0.00	9.86×10^{-05}	1.22×10^{-02}	4.90×10^{-01}
Streptococcus satellite phage Javan111	0.00	0.00	1.64×10^{-04}	1.22×10^{-02}	4.90×10^{-01}
Streptococcus satellite phage Javan304	0.00	0.00	1.00×10^{-04}	1.22×10^{-02}	4.90×10^{-01}
Streptococcus satellite phage Javan314	0.00	0.00	3.68×10^{-05}	1.22×10^{-02}	4.90×10^{-01}
Phage NCTB	2.01×10^{-05}	0.00	0.00	1.23×10^{-02}	4.90×10^{-01}
Phage 16	0.00	6.68×10^{-06}	1.37×10^{-03}	1.35×10^{-02}	5.26×10^{-01}
Faecalibacterium virus Taranis	5.33×10^{-03}	3.50×10^{-03}	1.56×10^{-03}	1.49×10^{-02}	5.41×10^{-01}
Parabacteroides phage YZ-2015b	3.23×10^{-04}	2.63×10^{-05}	1.94×10^{-04}	1.51×10^{-02}	5.41×10^{-01}
Bacteroides phage crAss001	3.61×10^{-04}	2.61×10^{-02}	3.56×10^{-05}	1.52×10^{-02}	5.41×10^{-01}
Edwardsiella virus pEtSU	1.55×10^{-04}	4.08×10^{-05}	6.30×10^{-05}	1.56×10^{-02}	5.41×10^{-01}
Streptococcus phage CHPC879	0.00	8.77×10^{-07}	9.20×10^{-05}	1.69×10^{-02}	5.41×10^{-01}
Streptococcus phage IPP24	0.00	3.02×10^{-06}	1.39×10^{-04}	1.69×10^{-02}	5.41×10^{-01}
Streptococcus phage Javan427	0.00	3.86×10^{-06}	2.33×10^{-04}	1.69×10^{-02}	5.41×10^{-01}
Streptococcus phage Javan83	0.00	2.33×10^{-05}	2.27×10^{-04}	1.95×10^{-02}	5.41×10^{-01}
Phage FAKO27_000238F	1.40×10^{-02}	1.17×10^{-04}	8.88×10^{-05}	2.41×10^{-02}	5.41×10^{-01}
Streptococcus phage YMC-2011	1.89×10^{-04}	5.51×10^{-05}	2.84×10^{-03}	2.69×10^{-02}	5.41×10^{-01}
Flavobacterium phage vB_FspS_laban6-1	1.42×10^{-05}	0.00	0.00	3.08×10^{-02}	5.41×10^{-01}

Gordonia phage CherryonLim	1.82×10^{-05}	0.00	0.00	3.08×10^{-02}	5.41×10^{-01}
Enterobacteria phage HK225	0.00	3.14×10^{-05}	0.00	3.22×10^{-02}	5.41×10^{-01}
Streptococcus phage IPP26	0.00	1.43×10^{-05}	0.00	3.22×10^{-02}	5.41×10^{-01}
Streptococcus phage Javan362	2.19×10^{-06}	2.11×10^{-04}	1.95×10^{-04}	3.27×10^{-02}	5.41×10^{-01}
Mycobacterium virus Trixie	2.02×10^{-05}	3.29×10^{-06}	1.93×10^{-06}	3.30×10^{-02}	5.41×10^{-01}
Prokaryotic dsDNA virus sp.	7.68×10^{-04}	5.11×10^{-04}	4.76×10^{-04}	3.63×10^{-02}	5.41×10^{-01}
Streptococcus phage Javan367	1.67×10^{-06}	1.75×10^{-04}	3.22×10^{-04}	3.83×10^{-02}	5.41×10^{-01}
Streptococcus phage P7574	0.00	1.73×10^{-05}	7.34×10^{-05}	3.86×10^{-02}	5.41×10^{-01}
Streptococcus satellite phage Javan243	5.38×10^{-06}	3.45×10^{-05}	3.92×10^{-04}	3.97×10^{-02}	5.41×10^{-01}
Siphoviridae sp.	2.23×10^{-04}	8.73×10^{-04}	1.08×10^{-04}	4.22×10^{-02}	5.41×10^{-01}
Streptococcus virus O1205	9.55×10^{-06}	0.00	9.94×10^{-05}	4.26×10^{-02}	5.41×10^{-01}
Streptococcus phage Javan115	1.05×10^{-06}	8.64×10^{-06}	4.15×10^{-04}	4.32×10^{-02}	5.41×10^{-01}
Caulobacter phage Ccr29	8.68×10^{-05}	0.00	2.77×10^{-05}	4.48×10^{-02}	5.41×10^{-01}
Streptococcus phage CHPC663	0.00	1.36×10^{-05}	3.10×10^{-04}	4.79×10^{-02}	5.41×10^{-01}
CRESS virus SC_1_H1_2017	8.03×10^{-05}	1.46×10^{-03}	3.03×10^{-05}	4.85×10^{-02}	5.41×10^{-01}
Streptococcus phage Javan345	7.26×10^{-05}	1.04×10^{-05}	1.44×10^{-04}	4.86×10^{-02}	5.41×10^{-01}

Note: Using the Kruskal-Wallis test to identify the differentially expressed viral species among control, adenoma, and CRC groups. *P*-adj was obtained by BH correction of *P* value.

Supplementary Table S7. Viral species with statistical differences between two comparisons

feature	Adenoma vs Control	CRC vs Control	CRC vs Adenoma
Phage FAKO27_000271F	↓	↓	/
Faecalibacterium virus Lugh	↓	↓	/
Faecalibacterium virus Toutatis	↓	↓	/
Faecalibacterium virus Oengus	↓	↓	/
uncultured phage	↓	↓	/
uncultured Caudovirales phage	↓	↓	/
Microvirus sp.	↓	↓	/
Bacillus virus G	↓	↓	/
Faecalibacterium virus Brigit	/	↓	/
Streptococcus phage CHPC1042	NA	↑	↑
Streptococcus satellite phage Javan434	NA	↑	↑
unidentified virus	NA	↑	↑
CrAssphage sp.	↓	↓	/
Faecalibacterium virus Epona	↓	↓	/
Acidovorax virus ACP17	↓	↓	NA
Streptomyces phage Kardashian	↓	↓	NA
Streptococcus phage Javan326	/	↑	/
Streptococcus phage Javan316	/	↑	↑
Streptococcus phage Javan191	/	↓	/
Streptococcus phage Javan110	/	↑	↑
Streptococcus phage Javan59	↑	↑	/
Erwinia virus Wellington	↑	↑	/
Streptococcus satellite phage Javan540	/	↑	↑
Microviridae sp.	↓	↓	/
Streptococcus phage Javan318	/	↑	/
Streptococcus phage IPP25	NA	↑	↑
Streptococcus phage IPP27	NA	↑	↑
Streptococcus satellite phage Javan111	NA	↑	↑
Streptococcus satellite phage Javan304	NA	↑	↑
Streptococcus satellite phage Javan314	NA	↑	↑
Phage NCTB	↓	↓	NA
Phage 16	/	↑	/
Faecalibacterium virus Taranis	/	↓	/
Parabacteroides phage YZ-2015b	↓	/	/
Bacteroides phage crAss001	↓	↓	/
Edwardsiella virus pEtSU	↓	/	/
Streptococcus phage CHPC879	/	↑	/
Streptococcus phage IPP24	/	↑	/
Streptococcus phage Javan427	/	↑	/
Streptococcus phage Javan83	/	↑	/
Phage FAKO27_000238F	↓	/	/
Streptococcus phage YMC-2011	/	↑	↑
Flavobacterium phage vB_FspS_laban6-1	/	/	NA

Gordonia phage CherryonLim	/	/	NA
Enterobacteria phage HK225	/	NA	/
Streptococcus phage IPP26	/	NA	/
Streptococcus phage Javan362	/	↑	/
Mycobacterium virus Trixie	/	↓	/
Prokaryotic dsDNA virus sp.	↓	↓	/
Streptococcus phage Javan367	/	↑	/
Streptococcus phage P7574	/	↑	/
Streptococcus satellite phage Javan243	/	/	↑
Siphoviridae sp.	↓	↓	/
Streptococcus virus O1205	/	/	↑
Streptococcus phage Javan115	/	↑	/
Caulobacter phage Ccr29	↓	/	↑
Streptococcus phage CHPC663	↑	↑	/
CRESS virus SC_1_H1_2017	/	↓	/
Streptococcus phage Javan345	/	/	↑

Note: Wilcoxon rank sum test and BH correction were used for comparison between every two groups. Viral species that are statistically different between the two groups compared with Padj values < 0.05. ↑ indicates a significant increase in the pre-vs group compared to the post-vs group. ↓ indicates a significant decrease in the pre-vs group compared to the post-vs group. / indicates no statistical difference. NA indicates that the species was not annotated in all samples of a given group in the two groups being compared.
